# Genetic Susceptibility to Joint Occurrence of Polycystic Ovary Syndrome and Hashimoto’s Thyroiditis: How Far Is Our Understanding?

**DOI:** 10.3389/fimmu.2021.606620

**Published:** 2021-02-26

**Authors:** Natalia Zeber-Lubecka, Ewa E. Hennig

**Affiliations:** ^1^ Department of Gastroenterology, Hepatology and Clinical Oncology, Centre of Postgraduate Medical Education, Warsaw, Poland; ^2^ Department of Genetics, Maria Skłodowska-Curie National Research Institute of Oncology, Warsaw, Poland

**Keywords:** autoimmune thyroid disease, Hashimoto’s thyroiditis, polycystic ovary syndrome, genetic variants, association studies, susceptibility loci

## Abstract

Polycystic ovary syndrome (PCOS) and Hashimoto’s thyroiditis (HT) are endocrine disorders that commonly occur among young women. A higher prevalence of HT in women with PCOS, relative to healthy individuals, is observed consistently. Combined occurrence of both diseases is associated with a higher risk of severe metabolic and reproductive complications. Genetic factors strongly impact the pathogenesis of both PCOS and HT and several susceptibility loci associated with a higher risk of both disorders have been identified. Furthermore, some candidate gene polymorphisms are thought to be functionally relevant; however, few genetic variants are proposed to be causally associated with the incidence of both disorders together.

## Introduction

Polycystic ovary syndrome (PCOS) is the most common endocrine disorder among women of reproductive age (1). Similarly, thyroid dysfunction, including Hashimoto’s thyroiditis (HT) as the principal autoimmune thyroid disease (AITD), is frequently observed among young women (2, 3). Both PCOS and HT can result in a wide range of metabolic syndrome features, including weight gain, impaired glucose tolerance, insulin resistance (IR), and dyslipidemia, which can lead to obesity, diabetes, or cardiovascular disease over a lifetime (4–6). Moreover, both disorders are major causes of infertility, a medical problem of a growing prevalence that is associated with strong physical, emotional, and socioeconomic consequences.

There is growing evidence that there may be mutual interaction between PCOS and HT. More importantly, combined occurrence of both diseases is associated with a higher risk of severe metabolic and reproductive complications than either PCOS or HT alone, and the severity of disease symptoms depends on the duration of the thyroid dysfunction (7–10). In addition, co-occurrence of thyroid autoimmune disease is associated with poorer response to treatment in infertile women with PCOS (11). The strong genetic influence on the development of these two diseases is well documented. Many candidate gene variants and susceptibility loci have been identified that are associated with a higher risk of PCOS or HT; however, the mechanisms underlying the close relationship between these two diseases, and common components to their genetic backgrounds, remain uncertain. Here, we comprehensively review a wide range of reports on genetic discoveries related to PCOS, HT, and their combined occurrence.

## Polycystic Ovary Syndrome

PCOS is a heterogeneous endocrinopathy that affects 5–20% of women of reproductive age (12). It is characterized mainly by clinical and/or biochemical hyperandrogenism (HA), ovary dysfunction (OD), often reflected by chronic oligo- or anovulatory menstrual cycles, and polycystic ovarian morphology (PCOM) on pelvic ultrasound; for diagnosis, two of these three criteria must be present (13). Consequently, four main PCOS phenotypes have been proposed: phenotype A, with HA, OD, and PCOM; phenotype B, with HA and OD but without PCOM; phenotype C, with HA and PCOM but without OD; and phenotype D, with OD and PCOM but without HA; with phenotypes A and B comprising an estimated > 40% of cases (14, 15). PCOS is characterized by symptoms of hypothalamic-pituitary-ovarian axis dysfunction, including increased hypothalamic gonadotropin-releasing hormone (GnRH) pulse frequency and GnRH quantity, as well as an elevated ratio of luteinizing hormone (LH) to follicle-stimulating hormone (FSH), which contribute to excessive ovarian androgen production and ovulatory dysfunction (16, 17).

Depending on the phenotype, POCS is associated with variable degrees of reproductive and metabolic dysfunction. Dyslipidemia occurs with a particularly high prevalence of up to 70% (14, 18). Also, defects in insulin activity and secretion, which may lead to IR, hyperinsulinemia, and impaired glucose tolerance, are observed very frequently (19, 20). PCOS is a low-grade inflammatory state (21); women with PCOS have higher levels of inflammatory markers such as C reactive protein (CRP), tumor necrosis factor α (TNF-α), and interleukin 6 (IL6), independent of associated obesity (22). Over a lifetime, PCOS is associated with a higher risk of menstrual irregularities, hirsutism, anovulatory infertility, spontaneous abortion, obesity, and obesity-linked co-morbidities such as nonalcoholic fatty liver disease and metabolic syndrome, type 2 diabetes mellitus, and cardiovascular pathologies, in addition to endometrial adenocarcinoma (20, 23–27). PCOS reduces quality of life, resulting in more frequent depressive episodes and suicide in women affected by this disease (28).

The etiology of PCOS is not fully elucidated. It is a complex disorder that might be represented by a common clinical phenotype of different processes. An IR or intrinsic ovarian dysfunction, possibly stimulated by an imbalance of female sex hormones, are considered inciting factors, which primarily results in increased androgen biosynthesis and secretion by the ovary (29, 30). Resulted hyperandrogenism can be further modulated by impaired insulin action (14). Insulin can also directly stimulate androgen production by amplifying theca cell responses to LH (31).

### Genetics of Polycystic Ovary Syndrome

PCOS is a complex, multifactorial disorder with a strong heritable component, as indicated by familial clustering and twin studies, which accounts for as much as 72% of the risk of syndrome development (32, 33). Until recently, the genomic approach to PCOS research was dominated by candidate gene association studies, including more than 100 genes whose alterations may be functionally involved in PCOS development. These genes are mainly related to the insulin signaling pathway and IR, chronic inflammation, reproductive hormone disorders, and biosynthesis of androgens; such as those encoding insulin (INS), INS receptor (INSR), INSR substrates (IRS), methylenetetrahydrofolate reductase (MTHFR), IL family members, TNF-α, toll-like receptor 2 (TLR2), fat mass and obesity-associated protein (FTO), paraoxonase 1 (PON1), calpain 10 (CAPN10), peroxisome proliferator-activated receptor γ (PPAR-γ), FSH receptor (FSHR), LH/choriogonadotropin receptor (LHCGR), LH-β, sex hormone-binding globulin (SHBG), androgen receptor, transforming growth factor β (TGF-β), fibrillin 3 (FBN3), and vitamin D receptor (VDR) (17, 33–37). Nevertheless, the vast majority of candidate gene variants associated with PCOS in previous studies have not been replicated, indicating the possibility of substantial genetic heterogeneity across various ethnic populations or different genetic architectures underlying specific PCOS phenotypes (38–49).

### Genome-Wide Association Studies for Polycystic Ovary Syndrome

To more deeply investigate genetic predisposition to PCOS, several genome-wide association studies (GWAS) have been carried out (**Table 1**) (50–54, 59, 66). The first two studies were conducted in China, and together identified 11 susceptibility loci located within or near genes implicated in: 1) gonadotropin activity, ovulation, and erectile dysfunction [*FSHR* and *LHCGR*, both at 2p16.3, and the zinc aminopeptidase gene (*C9ORF3*) at 9q22.32]; 2) insulin signaling and diabetes mellitus [the thyroid adenoma-associated protein (*THADA*) at 2p21, high mobility group AT-hook protein (*HMGA2*) at 12q14.3, and *INSR* at 19p13.3]; 3) organ growth control, cell proliferation, and apoptosis [yes-associated protein (*YAP1*) at 11q22.1 and zinc finger protein 217 (*ZNF217*) at 20q13.2]; 4) endosomal membrane trafficking and exocytosis of gonadotropins [DENN domain-containing protein 1A (*DENND1A*) at 9q33.3 and RAS-related protein (*RAB5B*) at 12q13.2]; and 5) modification of chromatin structure [TOX high mobility group box family member 3 protein (*TOX3*) at 16q12.1] (51, 52). Subsequently, associations between the single nucleotide polymorphism (SNP) rs13425728 in *LHCGR* with higher levels of testosterone, triglycerides, and low density lipoprotein (LDL) were detected among Hui Chinese subjects (56), and the two SNPs rs10818854 and rs10986105 in *DENND1A* were associated with increased HOMA-IR and anti-Müllerian hormone (AMH) levels in Arab women with PCOS (68).

**Table 1:** GWAS and replication studies for PCOS susceptibility.

Locus	Nearby genes	dbSNP ID^a/^		GWAS population [references]	Replication study population [references]	
					Replicated	Not replicated
1p22	OLFM3	rs11164278	*downstream*	KRN (50)		
**2p16.3**	**LHCGR**	rs13405728	*intron*	CHN (51, 52)	KRN (50, 53); EUR (54, 55); CHN (56–58)	EUR (59–63)
**2p16.3**	**FSHR**	rs2268361	*intron*	CHN (52)	KRN (50); EUR (54, 59, 64)	CHN (58, 65)
		rs2349415	*intron*	CHN (52)	CHN (65)	EUR (64); CHN (58)
**2p21**	**THADA**	rs13429458	*intron*	CHN (51, 52)	KRN (50, 53); EUR (59); CHN (57)	EUR (55, 60–64); CHN (56)
		rs12478601	*intron*	CHN (51)	KRN (53); EUR (54, 55, 63)	EUR (60, 64)
		rs12468394	*intron*	CHN (51)	KRN (53); EUR (54, 55, 63, 64)	EUR (60)
		rs7563201	*intron*	EUR (59, 66)		
2q34	ERRB4	rs1351592	*intron*	EUR (59)		
		rs2178575	*intron*	EUR (66)		
3q26.33	PEX5L	rs7652876	*upstream*	KRN (50)		
4p12	GABRB1	rs1159315	*intron*	KRN (50)		
4q35.2	TRIML1/TRIML2	rs7666129	*upstream*	KRN (50)		
5q31.1	RAD50	rs13164856	*downstream*	EUR (59, 66)		
8q24.2	KHDRBS3	rs10505648	*upstream*	KRN (50)		EUR (54)
8q32.1	GATA4/NEIL2	rs804279	*between*	EUR (54, 66)		
9p24.1	PLGRKT	rs10739076	*upstream*	EUR (66)		
9q21.32	RASEF	rs11536913	*upstream*	KRN (50)		
**9q22.32**	**AOPEP (C9orf3)**	rs3802457	*intron*	CHN (52)	EUR (54); CHN (58, 65)	EUR (59)
		rs4385527	*intron*	CHN (52)	EUR (54, 55)	KRN (50); CHN (58, 65); EUR (64)
		rs10993397	*intron*	EUR (54)		
		rs7864171	*intron*	EUR (66)		
**9q33.3**	**DENND1A**	rs2479106	*intron*	CHN (51, 52)	ASN (67)	KRN (50); CHN (56, 58); EUR (55, 59–64, 67); ARB (68, 69)
		rs10818854	*intron*	CHN (51)	KRN (53); ASN (67, 70); ARB (68); EUR (54, 55, 60, 63, 67, 70)	ARB (69); CHN (58)
		rs10986105	*intron*	CHN (51)	KRN (53); ASN (67); ARB (68); EUR (54, 55, 60, 64, 67)	ARB (69)
		rs9696009	*intron*	EUR (66)		
11p13	HIPK3	rs4755571	*3’UTR*	KRN (50)		
11p14.1	FSHB	rs11031006	*downstream*	EUR (54, 59)	CHN (71)	
		rs11031005	*downstream*	EUR (66)		
**11q22.1**	**YAP1**	rs1894116	*intron*	CHN (52)	KRN (50); EUR (54, 55, 59)	CHN (65)
		rs11225154	*intron*	EUR (59, 66)		
11q23.2	ZBTB16	rs1784692	*intron*	EUR (66)		
12p12.2	GYS2	rs7485509	*intron*	KRN (53)		
		rs10841843	*intron*	KRN (53)		
		rs6487237	*intron*	KRN (53)		
**12q13.2**	**RAB5B/SUOX**	rs705702	*5’UTR*	CHN (52)	KRN (50); EUR (54, 55, 59)	CHN (58, 65)
12q13.2	ERBB3	rs2271194	*intron*	EUR (66)		
12q14.3	HMGA2	rs2272046	*intron*	CHN (52)	EUR (59)	EUR (54, 64); CHN (58, 65)
12q21.2	KRR1	rs1275468	*upstream*	EUR (59)		
		rs1795379	*upstream*	EUR (66)		
**16q12.1**	**TOX3**	rs4784165	*downstream*	CHN (52)	KRN (50); EUR (59)	EUR (54); CHN (58, 65)
		rs8043701	*downstream*	EUR (66)		
16p13.3	SOX8	rs500492	*upstream*	KRN (50)		
19p13.3	INSR	rs2059807	*intron*	CHN (52)	EUR (64)	KRN (50); EUR (54, 55, 59); CHN (58, 65)
20q11.21	MAPRE1	rs853854	*intron*	EUR (66)		
20q13.2	ZNF217	rs6022786	*upstream*	CHN (52)	KRN (50)	EUR (54, 59); CHN (58, 65)

Bolded are loci replicated in at least two replication studies. Study populations: ARB, Arabic; ASN, Asian; CHN, Chinese; EUR, European; KRN, Korean.

a/ SNP identiﬁer based on NCBI SNP database (http://www.ncbi.nlm.nih.gov/snp/).

Later, two GWAS were conducted in Korean populations (50, 53). In the first, three novel SNPs in the glycogen synthase 2 (*GYS2*) gene on chromosome 12p12.2 were identified as being associated with PCOS through investigation of individuals with obesity-related condition (53); however, none of these variants reached the genome-wide significance threshold. In the second study, eight new susceptibility loci were suggested, including a main association signal at rs10505648 on chromosome 8q24.2, close to KH RNA-binding domain-containing, signal transduction associated 3 (*KHDRBS3*), which is associated with regulation of telomerase activity and variant splicing (72), and seven moderate signals at 1p22, 3q26.33, 4p12, 4q35.2, 9q21.32, 11p13, and 16p13.3 (50). In addition, eight of the Chinese susceptibility loci were replicated in Korean women (**Table 1**).

Two GWAS were recently conducted in populations of European ancestry, confirming the association of the three Asian susceptibility loci (*YAP1, THADA*, and *C9ORF3*), as well as identifying five novel loci corresponding to the epidermal growth factor receptor (*ERBB4/HER4*) gene at 2q34, double strand break repair protein (*RAD50*) at 5q31.1, the *GATA4/NEIL2* region encoding zinc finger transcription factor and endonuclease VIII-like 2 at 8q23.1, FSH β polypeptide (*FSHB*) at 11p14.1, and *KRR1* at 12q21.2, which encodes a ribosome assembly factor (**Table 1**) (54, 59). The same rs11031006 variant in the *FSHB* region, which was identified independently in both European GWAS, was associated with lower level of FSH and higher levels of LH and the LH/FSH ratio, suggesting that it may act by affecting gonadotropin secretion (54, 59). Together with the Chinese findings implicating *FSHR* variants, these results strongly suggest an etiologic role for gonadotropins in PCOS development. Additionally, the identified associations point to gonadal development, ovarian folliculogenesis, and follicular maturation, as well as repair of DNA damage, as processes involved in PCOS pathogenesis in European woman.

Recently, a large-scale genome-wide meta-analysis including 10,074 PCOS cases and 103,164 controls of European ancestry (66), confirmed 11 previously reported loci and identified three additional novel susceptibility loci: *ZBTB16* (zinc finger and BTB domain-containing 16) at 11q23.2, which is involved in cell cycle progression, control of the early stages of spermatogenesis, and endometrial stromal cell decidualization (73, 74); *MAPRE1* (microtubule associated protein RP/EB family member 1) at 20q11.20, which is involved in the regulation of microtubule structures and chromosome stability, and may participate in follicle development; and the plasminogen receptor (*PLGRKT*) gene at 9p24.1, which contributes to regulation of inflammatory responses and matrix metalloproteinase activation (**Table 1**). These newly identified loci provide additional evidence for the involvement of neuroendocrine, metabolic, and reproductive factors in PCOS. Additionally, the data suggest that the genetic architecture underlying the different PCOS phenotypes does not differ in terms of common susceptibility variants (66).

### Post-Genome-Wide Association Studies for Polycystic Ovary Syndrome

A number of gene polymorphisms discovered in Chinese populations, including *LHCGR*, *FSHR*, *THADA*, *DENND1A*, *C9ORF3*, *YAP1*, and *RAB5B/SUOX*, have been confirmed in at least two replication studies or meta-analyses for different Caucasian populations, and can be considered general PCOS susceptibility variants (**Table 1**) (55–58, 60–65, 67–71). However, GWAS have identified < 10% of the estimated heritability of PCOS (36), and there is relatively little overlap between the gene loci identified by candidate gene studies and those found by GWAS, likely because polymorphisms in gene coding regions are usually rare genetic variants that have a large impact on disease risk, whereas GWAS is designed to focus primarily on common variants with a low impact on genetic risk.

The greatest challenge for genomic studies is to identify causative mechanisms and determine the functional relevance of loci identified as risk factors by GWAS. A pathway-based approach has been proposed to increase the power to determine the biological meaning of GWAS results. Application of meta-analysis gene-set enrichment of variant associations (MAGENTA) to the PCOS GWAS dataset revealed oocyte meiosis and regulation of insulin secretion by acetylcholine and free fatty acids as biological pathways significantly associated with the syndrome (75). Among significantly associated genes, the *INS* gene was indicated in all three pathways. However, some caution should be taken with regard to these results as the analysis included variants of lower significance level than generally accepted for GWAS.

The genetic loci identified by GWAS are often named according to the nearest gene. While the nearby location of a gene does not necessarily mean that it is an effector gene at a given locus, several putative susceptibility genes located within GWAS-discovered risk loci for PCOS were found to be abnormally expressed in women with this condition. PCOS subtypes have been proposed based on phenotypes, expression quantitative trait loci, and biological pathway analyses, in which genes related to gonadotropin levels, metabolic mechanisms, or inflammation and its consequences, have independent impacts on the risk of syndrome development (76). Similarly, expression analysis of genes from 11 GWAS-identified PCOS risk loci revealed that different mechanisms may be involved in the pathogenesis of clinically diverse PCOS subtypes in relation to obesity, since *LHCGR* is over-expressed in non-obese women and *INSR* is under-expressed only in obese women with PCOS. A causal role was hypothesized for SNPs in the *LHCGR, INSR*, and *RAB5B* regions, suggesting that they may affect gene expression directly or indirectly through epigenetic mechanisms (77). Also, lower expression levels of the *RAB5A* gene in granulosa cells of obese patients with PCOS were observed relative to those in obese women without the syndrome, which may explain the high FSHR levels and FSH-FSHR signaling pathway disorders in PCOS (78). In turn, *HMGA2*, another gene identified by GWAS as a candidate, is expressed at high levels in granulosa cells of women with PCOS, where the *HMGA2/IMP2* pathway is suggested to be responsible for granulosa cell proliferation (79).

One of the most extensively studied GWAS-identified PCOS candidate genes is *DENND1A*, which encodes a clathrin binding protein (80). The PCOS risk SNP, rs10986105, in *DENND1A* was also found to increase the risk of hyperandrogenism in women without PCOS (60). DENND1A variant 2 (DENND1A.V2), a truncated splice isoform of DENND1A with a unique C-terminal sequence, is up-regulated in PCOS theca cells, and compelled expression of V2 in normal theca cells results in increased expression of genes encoding cytochrome P450 isoforms (*CYP17A1* and *CYP11A1*) and higher androgen production (81, 82). Recently, a functional network, including miR-130b-3p, DENND1A.V.2, LHCGR, RSB5B, and the signaling pathways they target, was proposed to potentially mediate PCOS-related hyperandrogenemia. The functional significance of this network is strongly supported by the discovery of the co-localization of LHCGR, DENND1A.V.2, and RAB5B proteins in theca cells (82), as well as the fact that decreased expression of miR-130b-3p, which is predicted to target DENND1A, correlates with increased V2 mRNA levels and androgen synthesis in PCOS theca cells (83).

Despite the clear and strong influence of genetic factors in the pathogenesis of PCOS, common genetic risk loci identified to date explain only a small percentage of estimated PCOS heritability. Therefore, next-generation sequencing studies to investigate entire genomic regions have been conducted to determine whether rare genetic variants with large effect size may contribute to PCOS pathogenesis. Potentially deleterious variants in *AMH* were identified using whole-genome and targeted sequencing of *AMH* in a case-control PCOS study (84). Seventeen PCOS-specific rare *AMH* coding variants resulted in significant reduction of AMH-mediated signaling in dual luciferase reporter tests. Therefore, *AMH* mutations in PCOS are suggested to result in decreased AMH-mediated inhibition of *CYP17* expression and androgen biosynthesis, leading to syndrome-specific hyperandrogenism. Furthermore, the missense variant rs104893836 in the first exon of the GnRH receptor gene (*GNRHR*) was detected by the whole-exome sequencing (85). Functional analyses revealed significantly reduced GnRH binding by the resulting variant GnRHR containing a Gln106Arg substitution; however, since this is a fairly common variant affecting the first extracellular receptor loop, its effect *in vivo* is likely to be mild (86). It is worth noting that an increase rather than a decrease in GnRH signaling is a hallmark of PCOS. Recently, whole-genome sequencing revealed several rare non-coding variants in *DENND1A* associated with reproductive and metabolic traits in PCOS families, suggesting their contribution to disease pathogenesis and providing additional evidence for the central role of DENND1A in PCOS (87).

## Autoimmune Thyroid Disease

AITD is the most common autoimmune disorder, affecting 5%–20% of the female population of fertile age (88). Its prevalence varies depending on age, geographical origin, and iodine intake (2). AITD is classified as an organ-specific autoimmune disorder mediated by T cells (89). An autoimmune attack, directed against components of the thyroid gland, may lead to clinically heterogeneous conditions, manifested by either thyroid hormone excess (hyperthyroidism), as in the case of Graves’ disease (GD), or reduced hormone production (hypothyroidism), a typical feature of HT, on which this review will mainly focus.

HT is considered the principal autoimmune disease among young women and the most frequent form of AITD, affecting 4%–9.5% of the adult population (90); its occurrence is eight times more common in women than in men (88). As a result of autoimmunity to self-antigens, approximately 60%–80% of patients with HT have serum antibodies against thyroglobulin (Tg) and 90%–95% have antibodies against thyroid peroxidase (TPO) (91, 92). HT is characterized by infiltration of lymphocytes and chronic inflammation of the thyroid gland, which promotes T cell-induced apoptosis of thyroid follicular cells. The Fas/Fas ligand (FasL) cascade is the main signaling pathway leading to apoptosis of thyroid cells in response to infiltration by pro-inflammatory cytokines, such as interferon (IFN)-γ, TNF-α, and IL1β (93). It is proposed that the mechanism of apoptosis leading to the characteristic thyroid destruction in HT may be a consequence of abnormal Fas/FasL regulation and decreased expression of the apoptosis regulator Bcl-2 (94, 95).

The progressive destruction, and finally fibrosis, of the glandular parenchyma often leads to hypothyroidism. AITD is considered the most frequent cause of hypothyroidism, although it can go unnoticed for years, without overt thyroid dysfunction. Subclinical hypothyroidism (SCH), defined as a serum thyroid-stimulating hormone (TSH) above the defined upper limit of the reference range, in combination with a normal level of free thyroxine (fT_4_), is more common than overt hypothyroidism, in which fT_4_ levels are reduced below the normal lower limit and TSH levels are further increased (96). HT has clinically heterogeneous presentation, from the presence of thyroid antibodies but normal thyroid function, to SCH or overt hypothyroidism. The prevalence of hypothyroidism among women with HT increases with age, and in most of cases it eventually develops despite patients initially being euthyroid (97). The diagnostic criteria for HT are based on detection of elevated levels of circulating anti-thyroid antibodies (anti-TPO and/or anti-Tg) and a typical hypoechogenic pattern of the thyroid gland on ultrasound examination (3). Although the presence of thyroid antibodies is a marker for thyroid damage, they are unlikely to play a role in the pathogenesis of HT (98).

Hypothyroidism may be associated with serious pregnancy complications, such as spontaneous abortion, preterm delivery, and placental abruption, as well as reduced fertility, ovulation disorders, insufficient endometrial thickness, and excessive, irregular menstrual bleeding (99–101). A recently published meta-analysis showed that SCH significantly increases the risk of miscarriage before 20 weeks of pregnancy and that early treatment can reduce the miscarriage rate (102). In hypothyroid states, lower levels of several hormones related to the ovarian axis, including SHBG, 17-β-estradiol (E_2_), testosterone, and androstenedione, were observed (103). As a result of increased secretion of thyrotropin-releasing hormone (TRH), prolactin levels can increase, while LH and FSH concentrations remain normal (104). Underactive thyroid is also associated with the formation of ovarian cysts, which were normalized after T_4_ replacement (105, 106).

Metabolic changes are also common in hypothyroidism, particularly dyslipidemia and IR, and the severity of these disorders correlates with TSH concentration, even within its normal range (107–109). A link between autoimmunity and obesity has been noted, with leptin as a factor linking the two conditions (110). Being overweight during childhood is positively associated with anti-TPO antibody levels in women at 60–64 years-of-age (111). Since thyroid hormones have a wide impact, the symptoms of thyroid dysfunction can be systemic, affecting almost every physiological system, in addition to significant reproductive or metabolic defects (3). Nevertheless, the pathogenesis of HT remains largely unknown.

### Genetics of Autoimmune Thyroid Disease

As in the case of PCOS, a strong genetic predisposition to AITD has been established, suggesting that over 70% of the susceptibility to the development of antibodies directed against thyroid antigens can be attributed to genetic factors (112). Among first-degree relatives of patients with HT, the risk of developing HT is increased by 20- to 30-fold; in 60% of first-degree relatives of patients with HT, anti-thyroid antibody positivity is observed (27). Also, the high concordance of HT in monozygotic twins (55%) strongly supports a genetic component in AITD predisposition (113).

Candidate gene case-control studies identified several putative susceptibility variants associated with AITD development or progression. These included variants in genes encoding proteins related to inflammation, modulation of immune responses, or specific for the thyroid, such as: human leukocyte antigen (HLA) class I and class II, forkhead box P3 (FOXP3), cytotoxic T-lymphocyte-associated protein 4 (CTLA4), cluster of differentiation 40 (CD40), protein tyrosine phosphatase, non-receptor type 22 (PTPN22), selenoprotein S (SEPS1), IL4, IL2 receptor α (IL2RA), VDR, Tg, TSH receptor (TSHR), signal transducer and activator of transcription 3 (STAT3), and STAT4, with *HLA-DR3* carrying the highest risk (2, 114–120). Among non-HLA genes, *CTLA4* and *PTPN22* were most consistently identified as predisposing to both HT and GD (121–124), while the *TSHR* locus appears to be specific for GD, but not HT, suggesting some genetic differences between these two types of AITD (125, 126); however, similar to PCOS, candidate gene screening for HT susceptibility mainly generated controversial and non-replicable findings (127–132). Limited understanding of disease pathogenesis and the consequent lack of comprehensive datasets on candidate genes, small sample sizes, and low statistical power, were some of the main limitations of candidate gene studies for both disorders. The era of GWAS and subsequent replication studies have brought further data on genetic susceptibility to HT.

### Genome-Wide Association Studies and Post-Genome-Wide Association Studies for Autoimmune Thyroid Disease and Thyroid Function Related Traits

Several GWAS for AITD (HT or GD), hypothyroidism, positivity for anti-thyroid (anti-TPO or anti-Tg) antibodies, and thyroid function parameters (including TSH or fT_4_ levels), have been undertaken (**Table 2**) (133–135, 139–146, 148, 154, 155, 157–160). Some GWAS-identified AITD susceptibility loci have been replicated in different populations (**Table 2**) (136–138, 147, 149–153, 156); however, HT is rather poorly represented among GWAS. Numerous studies have been performed using small cohorts or patients with HT and GD grouped together (128, 161). In total, six GWAS included separate groups of patients with HT (134, 140, 146, 147, 154, 159), while three included patients with hypothyroidism, a typical feature of HT (133, 135, 158). Together, 16 putative HT susceptibility loci have been identified; however, the most convincing evidence for associations is limited to *HLA* (6p21), *CTLA4* (2q33.2), *PTPN22* (1q13.2), and *FOXE1* (9q22.33) variants. Additionally, two loci*, TPO* (2p25.3) and *ATXN2* (ataxin2; 12p24.12), are convincingly associated with the presence of anti-TPO antibodies, and three loci, *CAPZB* (capping actin protein of muscle Z-line subunit beta; 1p36.13), phosphodiesterase *PDE8B* (5q13.3), and *PDE10A* (6q27), are associated with TSH levels (**Table 2**).

**Table 2:** GWAS and replication studies for AITD and thyroid dysfunction traits susceptibility.

Locus	Nearby genes	dbSNP ID^a/^	GWAS		Replication study	
			Population [references]	Phenotype	Population [references]	Phenotype
**1p13.2**	**PTPN22**	rs2476601 *(1858 C/T)*	USA-EUR (133) UK (134)	hypothyroidism HT+GD	EUR (135, 136) POL (137)	hypothyroidism HT, GD
		rs6679677	USA-EUR (133)	hypothyroidism		
		rs3811021			POL (138)	HT
1p13.2	MAGI3	rs1230666	EUR-CAU (139)	anti-TPO level		
1p13.3	VAV3	rs4915077	USA-EUR (133)	hypothyroidism		
		rs7537605	JAP (140)	HT		
		rs12126655	KRN (141)	TSH level		
**1p36.13**	**CAPZB**	rs1472565	USA-EUR (133)	hypothyroidism		
		rs10799824	ICE (142)	TSH level	USA-EUR (143)	TSH level
		rs10917469	UK (144)	TSH level		
		rs6683419			CHN (145)	TSH level
2p16.3	FSHR	rs12713034	CRT (146)	anti-TPO/Tg-positivity		
2p25.1	TRIB2	rs1534422	UK (134)	HT+GD		
**2p25.3**	**TPO**	rs11675434	EUR-CAU (139)	anti-TPO-positivity, anti-TPO level	CRT (147) CRT (148)	HT anti-TPO/Tg-positivity
		rs2071403	KRN (141)	anti-TPO-positivity		
		rs11211645			POL (138)	HT
**2q33.2**	**CTLA4**	rs231775 *(A49G)*	UK (134)	HT+GD	POL (149); IND (150)	HT, GD
		rs3087243 *(CT60)*	EUR (135)	hypothyroidism	EUR (136) JAP (151); CHN (152)	hypothyroidism HT, GD
		rs231779	USA-EUR (133)	hypothyroidism		
2q36	IGFBP5	rs13015993	USA-EUR (143)	TSH level		
		rs6435953			CRT (153)	anti-Tg level
3q13.12	DUBR	rs561030786	CRT (146)	anti-TPO/Tg-positivity		
3q21	KALRN	rs2010099	EUR-CAU (139)	anti-TPO level		
3q27.3	LPP	rs13093110	UK (134)	HT+GD		
4q27	GPR103	rs7679475	NHW (154)	HT		
		rs1513695	NHW (154)	HT		
4q31.2	NR3C2	rs10032216	USA-EUR (143)	TSH level		
		rs11935941			CRT (153)	HT
4q32.3	TRIM61 TRIM60	rs12507813	CRT (146)	anti-TPO-positivity		
**5q13.3**	**PDE8B**	rs4704397	ITN (155)	TSH level	USA-EUR (133) CRT (156)	hypothyroidism HT
		rs6885099	ITN (155)	TSH level	KRN (141); USA-EUR (143)	TSH level
		rs2046045	ICE (142)	TSH level		
5q21.2	RP11-138J23.1	rs13190616	CRT (146)	anti-TPO/Tg-positivity		
**6p21.3**	**HLA class I**	rs2517532 rs2516049	USA-EUR (133) USA-EUR (133)	hypothyroidism hypothyroidism	CRT (157)	HT
**6p21.3**	**HLA-DPB2**	rs733208	KRN (141)	anti-TPO-positivity		
**6p21.3**	**HLA-DRB1**	rs17886918			POL (138)	HT
**6p21.3**	**HLA-DQB1**	rs3210176			CRT (157)	HT
6p21.3	IP6K3	rs791903	CRT (157)	HT		
6q15	BACH2	rs72928038 rs10944479	UK (134) EUR-CAU (139)	HT+GD anti-TPO-positivity		
6q27	DLL1	rs4710782	CRT (148)	anti-Tg level		
6q27	PDE10A	rs2983521	ITN (155)	TSH level		
		rs3008043	ICE (142)	TSH level		
		rs753760			USA-EUR (143)	TSH level
7q31.31	ANKRD7 LSM8	rs6972286	CRT (146)	anti-Tg-positivity		
8q12.1	XKR4	rs2622590	CHN (145)	TSH level		
9p24	GLIS3	rs1571583 rs10974423	USA-EUR (143)	TSH level	CRT (153)	anti-TPO level
9q21.2	GNA14	rs75201096	CRT (157)	HT		
**9q22.33**	**FOXE1**	rs7850258	USA-EUR (158)	hypothyroidism	EUR (136)	hypothyroidism
		rs965513	USA-EUR (158) ICE (142)	hypothyroidism TSH level	CRT (157)	HT
		rs925489	USA-EUR (158)	hypothyroidism	USA-EUR (133) CHN (145)	hypothyroidism TSH level
9q31.1	GRIN3A	rs4457391 rs1935377	CRT (148) CRT (148)	anti-TPO/Tg level anti-TPO level		
9q32	WHRN	rs4979402			CRT (156)	anti-Tg level
11q21	FAM76B	rs4409785	UK (134)	HT+GD		
12q24.12	SH2B3	rs3184504	USA-EUR (133)	hypothyroidism	CRT (156)	HT, anti-TPO level
**12q24.12**	**ATXN2**	rs653178	EUR-CAU (139)	anti-TPO-positivity	CRT (148)	anti-TPO/Tg level
		rs10774625			CRT (147)	HT, anti-TPO-positivity
14q13	MBIP	rs1537424	USA-EUR (143)	TSH level	CRT (153)	HT, anti-Tg level
14q31	CEP128	rs327463	CHN (159)	HT, GD		
15q14	RASGRP1	rs7171171			CRT (147)	HT, anti-TPO-positivity
16q24	IRF8	rs16939945	CRT (160)	anti-TPO/Tg-positivity		
17q21.33	CA10	rs756763	CRT (146)	anti-Tg-positivity		
17q25	SDK2	rs12944194	CRT (157)	HT		
18p11.32	YES1	rs77284350	CRT (160)	anti-TPO/Tg-positivity		
19p13.2	INSR	rs4804416	USA-EUR (143)	TSH level	CRT (153)	anti-Tg level

Bolded are loci replicated in at least two replication studies. Study populations: ASN, Asian; CAU, Caucasian; CHN, Chinese; CRT, Croatian; EUR, European; ICE, Icelandic; IND, Indian; ITN, Italian; JAP, Japanese; KRN, Korean; NHW, non-Hispanic White; POL, Polish; EUR-CAU, EUR-Caucasian ancestry; USA-EUR, USA-European ancestry.

GD, Graves’ disease; HT, Hashimoto’s thyroiditis; Tg, thyroglobulin; TPO, thyroid peroxidase; TSH, thyroid-stimulating hormone.

a/ SNP identiﬁer based on NCBI SNP database (http://www.ncbi.nlm.nih.gov/snp/).

A genetic overlap was observed between susceptibility loci identified in GWAS for AITD or hypothyroidism and GWAS for thyroid function, including *FOXE1*, *CAPZB*, and *PDE8B*. *FOXE1*, also known as TTF-2 (thyroid transcription factor 2), is associated with hypothyroidism, and with TSH and fT_4_ levels, as well as with thyroid cancer (133, 143, 158, 162). This factor is involved in thyroid gland development and differentiation (163, 164). FOXE1 regulates *TG* and *TPO* transcription by binding to response elements in their promoter regions (165), and is necessary for synthesis of thyroid hormones. Both PDE8B and CAPZB also have strong links to thyroid function. *PDE8B* encodes a phosphodiesterase that catalyzes the hydrolysis of cAMP and is primarily expressed in the thyroid gland (166). It is proposed that PDE8B may affect TSH release from the pituitary and mediate the effects of TSH in the thyroid by altering cAMP levels (155). A role for PDE8B in cAMP-dependent generation of triiodothyronine (T_3_) and T_4_ has also been proposed (143). Similarly, CAPZB, a member of the F-actin-capping protein family, is highly expressed in thyroid tissue. As a protein associated with cytoskeleton remodeling and assembly of cytoplasmic microtubules, it may contribute to the disturbance of thyroid follicular architecture commonly observed in AITD.

Interestingly, three independent GWAS connected *VAV3* (vav guanine nucleotide exchange factor 3) variants with HT, hypothyroidism, and TSH levels (133, 140, 141). VAV3 activates Rho and Rac GTPases and is important for both thyroid and immune function (167). VAV3 is also necessary for B-cell receptor endocytosis and antigen presentation by major histocompatibility complex (MHC) class II molecules (168).

Notably, the MHC region, *CTLA4*, and *PTPN22*, which were identified by GWAS as most significantly associated with HT, were previously described as AITD susceptibility loci, based on a systematic review of candidate genes (128). All of these loci are related to autoimmune responses and involved in antigen presentation and T cell receptor signaling.

#### Human Leukocyte Antigen Alleles

The highly polymorphic MHC region of chromosome 6p21, encoding the HLA glycoproteins, is the most intensively studied region of the genome in the search for associations with development of AITD. Numerous *HLA* alleles have been identified as genetic risk factors for AITD; however, the data on *HLA* haplotypes in HT are less definitive than that in GD. Although, some *HLA* alleles associated with HT are common to GD, others appear to be unique to each disease (119, 169, 170), suggesting that *HLA* genotypes may contribute, at least in part, to differences in HT and GD immunopathogenic mechanisms.

Among the *HLA* class I alleles, HLA-A*02:07 was associated with HT susceptibility in a Japanese population (169, 170). Of note, this allele was the strongest and most significantly associated susceptibility allele in HT, in contrast to GD, where alleles from the MHC class II region were more strongly associated (169). Furthermore, it was estimated that approximately 58% of patients with HT carried at least one of four *HLA* class I alleles (*HLA*-A*02:07, B*35:01, B*40:02, or B*40:06) (169). Conversely, in Caucasian populations, only a few studies have suggested associations between *HLA* class I alleles and HT risk (171, 172). Hence, it is hypothesized that ethnic differences may play a role in the immunological mechanisms involved in thyrocyte destruction (169).

Numerous associations between *HLA* class II alleles and HT have been demonstrated (particularly in Caucasian populations), including DQA1*03, DQB1*02, DQB1*03, DRB1*03, and DRB1*04 alleles, or *DR3*, *DR4*, and *DR5* haplotypes (173–176). In Japanese population, DRB1*04 and DRB4*01 were suggested as susceptibility alleles, while DQA1*01 and DQB1*06 were protective (169). However, all of these studies included relatively small cohorts and generated inconsistent results. Some of these previous observations have been confirmed in a large group of UK Caucasian patients with HT; DRB1*04, DQB1*03:01, and DQA1*03:01 showed the most significant predisposing effect, while DQA1*01:02, DQA1*02:01, and DQB1*06 had the most significant protective effect. Overall, a strong predisposing association between *DR4* or *DR8* haplotypes and HT has been found, as well as a borderline association with *DR3*, whereas protective effects were detected for *DR13*, *DR7*, and *DR15* (119). Similarly, DRB1*04:05, DQB1*02:01, DQB1*03:02, and DQA1*03:01 allele frequencies were higher in Greek patients with HT, while those of DRB1*07 were lower, than in the control group (177).

In recent studies including non-Caucasian populations, the HLA-DRB4*53:01 allele was identified as associated with HT susceptibility in Japanese patients (170) and the *DR8* haplotype in Koreans (178). Additionally, *HLA* alleles in the *HP-2* haplotype (HLA-A*33:03-C*14:03-B*44:03-DRB1*13:02-DQB1*06:04-DPB1*04:01) and the *HP-2* haplotype itself were associated with protective effects against GD and HT in a Japanese population (169, 170), with OR = 0.36 for HT (169). Furthermore, the HLA-DRB1*03 allele was shown to be predisposing in Kayseri Turkish patients with HT, in contrast to DRB1*01, which was associated with a protective effect (179). Moreover, in an Indian population, DRB1*12 and DRB1*10 exhibited the strongest predisposing and protective effects, respectively, among different *HLA-DRB1* alleles (150); however, in contrast to previous reports, a decreased frequency of the DRB1*03 allele was observed in HT patients in this study.

Due to its crucial role in the presentation of peptide antigens to T cells, particular attention has been paid to the molecular structure of the peptide binding pocket in HLA class II molecules. Arginine at position 74 of the HLA-DRβ1 chain (DRβ1-Arg74) was identified as a critical pocket amino acid signature that confers susceptibility to both GD and HT (180, 181). Conversely, the presence of glutamine at position 74 of the DRβ1 chain conferred a protective effect. It was suggested that a specific HLA-DR peptide binding pocket structure may predispose to AITD by enabling presentation of certain autoantigens, such as Tg, TPO, or TSHR pathogenic peptides. Indeed, the non-synonymous *TG* variant, W1999R, can interact with HLA-DRβ1-Arg74, and together these two variants confer a high risk for AITD (182). Recently, among Polish patients, HLA-DRB1 with phenylalanine at position 67 (encoded by the rs17886918T variant) was significantly associated with HT (138).

#### Cytotoxic T-Lymphocyte-Associated Protein 4

GWAS identified several *CTLA4* genetic variants as being associated with AITD (both HT and GD) or hypothyroidism, and these associations were widely replicated in various ethnic groups (**Table 2**). The most consistent associations with HT were a SNP at position 49 (A49G, rs231775) in the CTLA4 leader peptide, resulting in an alanine to threonine substitution, and a SNP located near to the 3’ UTR (CT60, rs3087243) in both the Caucasian and Asian populations (**Table 2**). Several subsequent meta-analyses have confirmed these associations (183–187). *CTLA4* is suggested to contribute to AITD susceptibility by interacting with other loci (188) and a strong synergy has been demonstrated for the *CTLA4* and *HLA* genes in AITD (189). In an Indian population, associations with both susceptibility (for a combination of the G allele of *CTLA4*, A49G, and the *HLA-DR5* allele) and protection (for a combination of the A allele of *CTLA4*, A49G, and the *HLA-DR3*, *DR10*, and *DR15* alleles) have been established (150).

CTLA4 is a transmembrane protein expressed on activated T cells that functions as a negative regulator of their activation by competing with CD28 for binding to the ligand B7 on antigen-presenting cells (190). Therefore, it is likely that polymorphisms that reduce *CTLA4* expression or activity may cause excessive activation and proliferation of T cells, predisposing to autoimmunity (114, 191). Consistent with this, SNPs in *CTLA4* are risk variants for various autoimmune disorders (192); however, it is unclear which *CTLA4* variants are causative and by what mechanism they confer susceptibility to autoimmunity.

#### Protein Tyrosine Phosphatase, Non-Receptor Type 22

Similar to CTLA4, PTPN22 is a powerful inhibitor of T cell activity; it belongs to a family of protein tyrosine phosphatases expressed in both mature and immature B and T cells. PTPN22 binds to the SH3 domain of the C-terminal Src kinase (Csk), thereby suppressing kinases that mediate T cell signaling (193). PTPN22 also functions as a negative regulator of T cell activation through its interaction with the Grb2 adaptor molecule (194).

After the HLA system, *PTPN22* polymorphisms may be the most important genetic risk factor for autoimmune diseases (195). Variations in *PTPN22* are associated with various autoimmune disorders, including AITD (191). The most extensively studied and widely confirmed is the association of the C1858T missense mutation (rs2476601), which results in a substitution of arginine (R) to tryptophan (W) at position 620 (R620W) of the encoded protein. The *PTPN22* C1858T variant is associated with an increased risk of both HT and GD (196, 197); however, significant differences in this association have been observed across various ethnic groups. A significant association between the *PTPN22* C1858T polymorphism and susceptibility to AITD was demonstrated specifically in Caucasians, but it was hardly detected in non-Caucasian populations (198–201). It is suggested that this could be due to the ancestral effects and different prevalence rates of rare variants in the studied populations. Indeed, the susceptibility allele (T) of rs2476601 is generally extremely rare in Asian and African populations (198, 202). Also, a decreasing frequency of this allele was observed from the north to the south of the Europe (203). It is suggested that AITD susceptibility in different ethnic populations may be related to distinct risk loci. Notably, an extremely rare predisposing variant of *PTPN22* (missense A77G mutation) has recently been identified in a Chinese HT pedigree using whole-exome sequencing (204).

Although the exact mechanism by which the R620W variant predisposes to autoimmunity remains largely unknown, PTPN22 with a tryptophan residue at position 620 (Trp^620^) binds Csk less efficiently than the variant with an arginine at this position (205). Consequently, the capacity of PTPN22 to downregulate T cell responses may be reduced, thereby increasing susceptibility to autoimmunity. By contrast, PTPN22 Trp^620^ is more active and a more potent negative regulator of T cell signaling, suggesting a role for R620W in positive selection of autoreactive T cells (206). Furthermore, PTPN22 Trp^620^ was associated with increased frequencies of thymically-derived T regulatory (Treg) cells and with increased expression of programmed cell death protein (PD-1) on both Treg and T effector cells (207). Currently, the role of the *PTPN22* C1858T polymorphism in autoimmunity is suggested to be alteration of both the innate and adaptive immune responses (206, 208).

Overall, current knowledge of HT genetics is rather limited. The majority of identified associations relate to general immune-regulatory genes involved in the development of central and peripheral tolerance and antigen presentation, which are important for achieving the proper balance between an adequate immune response against foreign antigens and maintaining autoantigenic tolerance. Moreover, all susceptibility loci identified to date together account for only a small proportion of the heritability of HT (209). It is estimated that < 5.5% of total HT variance can be explained by common genetic variants (138, 157), indicating that a substantial number of HT predisposing factors remain to be discovered.

## Joint Prevalence of Polycystic Ovary Syndrome and Autoimmune Thyroid Disease

Considering the similar elements in the pathogenic mechanisms and high prevalence rates of both AITD and PCOS among women of reproductive age, the interesting question of whether thyroid dysfunction is significantly more frequent in women with PCOS arises. An increasing number of studies indicate a higher prevalence of AITD, and particularly hypothyroidism, in patients with PCOS (**Table 3** and relevant references therein). In most of these studies, although not all, higher levels of anti-TPO and/or anti-Tg antibodies were observed, exceeding the upper limit of the normal range in an average of 22.3% of patients with PCOS (ranging from 4.8% to 37.9%, depending on the study), compared with an average of 8.5% in healthy women (range 3.3% to 15.3%). Also, decreased echogenicity of the thyroid gland, a characteristic ultrasound pattern typical of HT, was observed more frequently in women with PCOS than in those without the condition (mean, 29% *vs.* 9%, respectively). The prevalence of SCH at TSH levels between 4.2 and 10 mIU/L ranged from 11.3% to 30.3% (mean, 20.3%) among patients with PCOS, which was more than 3 times higher than that reported for women without this condition (mean, 6.2%; range, 1.9% to 8.75%). Altogether, the estimated prevalence of AITD among women with PCOS was nearly threefold higher than that in healthy women (mean, 28% *vs.* 10%, respectively), although it should be noted that the diagnostic criteria for AITD differed among studies (**Table 3**).

**Table 3:** Joint prevalence of SCH and AITD in women with PCOS.

Study year (ref)	Compared groups	*N*	Population	TSH and thyroid hormone level	Prevalence of SCH;TSH cut-off value	Anti-TPO and anti-Tg antibody levels	Hypoechoic thyroid (goiter)	Prevalence of AITD; AITD criteria
2004 (210)	PCOS vs. age-matched women without PCOS	175 vs. 168	German	TSH 2.0 ± 1.0 vs. 1.4 ± 0.6 mIU/L *p*<0.001; TSH level above upper limit 10.9% vs. 1.8% *p*<0.001; fT_4_ level - NS		Anti-TPO and/or anti-Tg positivity 26.9% vs. 8.3% *p*<0.001	42.3% vs. 6.5% *p*<0.001	20.6% vs. 6.5% *p*<0.001; Anti-TPO and/or anti-Tg positivity and hypoechoic thyroid
2011 (211)	PCOS vs. age-matched healthy women	84 vs. 81	Turkish	TSH, fT_3_ and fT_4_ levels - NS	TSH > 4.5 mIU/L	Anti-TPO and anti-Tg levels - NS	Thyroid volume and heterogeneity of thyroid parenchyma - NS	PCOS alone was not associated with AITD; Higher thyroid volume (*p*=0.001), anti-TPO (*p*=0.005) and anti-Tg (p=0.003) levels in MS
2012 (212)	PCOS vs. age-matched healthy women	78 vs. 350	Iranian	TSH level - NS		Higher anti-TPO median level *p*=0.04;Anti-Tg level - NS	Goiter 62.3% vs. 35.7% *p*<0.0001	
2013 (213)	PCOS vs. age and BMI-matched women without PCOS	80 vs. 80	Indian	TSH 4.55 ± 2.66 vs. 2.67 ± 3.11 mIU/L *p*<0.05; Higher fT_3_, fT_4_ levels *p*<0.001	22.5 vs. 8.75% *p*<0.05; TSH > 4.25 mIU/L	Anti-TPO 28.04 ± 9.14 vs. 25.72 ± 8.27 IU/ml *p*=0.035	12.5% vs. 2.5% *p*<0.001; Goiter 27.5% vs. 7.5% *p*<0.001	22.5% vs. 1.25% *p*<0.05; Anti-TPO positivity
2013 (7)	PCOS infertile vs. infertile controls	151 vs. 155	Italian	TSH 2.17 ± 1.19 vs. 1.82 ± 1.1 mIU/L *p*<0.009; fT_3_, fT_4_ levels - NS	33.7% vs. 23.2% *p*<0.05; TSH > 2.5 mIU/L			
2013 (214)	PCOS vs. age-matched healthy women	113 vs. 100	Italian	TSH level above the normal range in 43.3% (13/30) of PCOS patients with AITD	SCH in 43.3% (13/30) of PCOS patients with AITD, remaining have normal thyroid function	Anti-TPO and anti-Tg positivity in 53.3% (16/30) of PCOS patients with AITD; Anti-TPO positivity in 33.3% (10/30) of PCOS patients with AITD	93.3% (28/30) of PCOS patients with AITD	27% vs. 8% *p*<0.001; At least two of three criteria: anti-TPO and/or anti-Tg positivity, TSH levels above the normal range and hypoechoic thyroid
2014 (215)	PCOS euthyroid vs. healthy women	56 vs. 30	Syrian	TSH and fT_4_ levels - NS	TSH > 4.2 mIU/L	Anti-TPO positivity 19.6% vs. 3.3% *p*<0.05; Anti-Tg positivity 21.4% vs. 3% *p*<0.05; Anti-TPO 39.9 ± 59.5 vs. 18.9 ± 11.2 IU/ml *p*=0.013; Anti-Tg level - NS		28.6% vs. 3.3% p<0.05; Anti-TPO and/or anti-Tg positivity
2015 (216)	PCOS vs. age-matched women without PCOS	73 vs. 60	Turkish	TSH and fT_4_ levels - NS		Anti-TPO and anti-Tg positivity - NS	Thyroid nodules frequency and thyroid gland volume - NS	32.5% vs. 23.3% - NS; Anti-TPO and anti-Tg positivity and/or hypoechoic thyroid
2015 (6)	PCOS vs. women without PCOS	65 vs. 65	Brazilian	TSH 2.9 ± 1.8 vs. 2.2 ± 1.2 mlU/L *p*=0.013; fT_3_ level *p*=0.002; fT_4_ level - NS	16.9% vs. 6.2% *p*<0.05*;* TSH > 4.5 mlU/L	Anti-TPO and anti-Tg positivity - NS	26.8% vs. 15.4% *p*=0.052	43.1% vs. 26.2% *p*=0.04; At least two of three criteria: anti-TPO and/or anti-Tg positivity, TSH levels above the normal range and hypoechoic thyroid
2015 (217)	PCOS vs. age-matched healthy women	64 vs. 68	Slovak	TSH and fT_4_ levels - NS	Hypothyroidism 10.94% vs. 13.24% - NS; TSH > 4.5 mIU/L	Anti-TPO positivity 18.75% vs. 7.35% *p*=0.045; Anti-Tg positivity - NS		18.75% vs. 10.29% - NS; Anti-TPO and/or anti-Tg positivity and/or hypoechoic thyroid
2015 (218)	PCOS vs. age-matched healthy women	142 vs. 52	Argentine	TSH 3.4 ± 2.8 vs. 1.8 ± 0.9 mlU/L p<0.001; fT_4_ level - NS	30.3% vs. 1.9% *p*<0.001; TSH ≥ 4.2 mIU/L	Anti-TPO positivity 19% vs. 13.5% - NS		36.6% vs. 13.5% *p*<0.001; Anti-TPO positivity and/or SCH
2015 (219)	PCOS vs. age and BMI-matched healthy women	86 vs. 60	Turkish	TSH median level 2.9 vs. 1.8 mlU/L *p*=0.037; TSH level above the normal range 26.7% vs. 5% *p*=0.001;fT_3_ and fT_4_ levels - NS	TSH > 4.25 mIU/L	Anti-TPO positivity 26.7% vs. 6.6% *p*=0.002; Anti-Tg positivity 16.2% vs. 5% *p*=0.039; Higher anti-TPO median level *p*=0.017; Higher anti-Tg median level *p*=0.014		22.1% vs. 5% *p*=0.004; Anti-TPO and/or anti-Tg positivity and hypoechoic thyroid
2016 (220)	PCOS vs. age and BMI-matched healthy women	100 vs. 100	Chinese	TSH 5.11 ± 22.2 vs. 2.9 ± .2 mlU/L *p*<0.001; fT_3_ level *p*=0.03; fT_4_ level - NS	27% vs. 8% *p*=0.0002; Overt hypothyroid 3% vs. 0% *p*=0.01; TSH > 4.25 mIU/L	Anti-TPO 76.2 ± 23.4 vs. 20.14 ± 12.4 IU/ml *p*<0.001	34% vs. 7% p<0.01; Goiter 25% vs. 2% *p*=0.02	25% vs. 2% *p*<0.001; Anti-TPO and hypoechoic thyroid
2016 (221)	PCOS vs. age-matched women without PCOS	55 vs. 51	Indian	TSH, fT_3_ and fT_4_ levels - NS	TSH > 6.2 mIU/L	Anti-TPO 49.54 ± 136.9 vs. 22.63 ± 38.5 IU/ml - NS; Higher anti-Tg level *p*=0.004		37.7% vs. 15.6% - NS; Anti-TPO and/or anti-Tg positivity
2017 (222)	PCOS vs. age-matched women without PCOS	90 vs. 90	Indian	TSH and fT_4_ levels - NS	6.6% vs. 5.6% - NS	Anti-TPO 25.8 ± 2.9 vs. 14.6% ± 2.3 5 IU/ml *p*<0.009		25% vs. 5.6% *p*<0.05; Anti-TPO positivity
2017 (223)	PCOS vs. age-matched healthy women	97 vs. 71	Turkish	TSH and fT_4_ levels - NS	TSH > 5. 33 mIU/L	Anti-TPO positivity 32.0% vs. 15.5% *p*=0.019; Anti-Tg positivity 16.5% vs. 5.6% *p*=0.051	Thyroid nodules 29.9% vs. 15.5% *p*=0.043	40.2% vs. 15.5% *p*=0.001; Anti-TPO and/or anti-Tg positivity and/or hypoechoic thyroid
2017 (224)	PCOS vs. normo-ovulatory, age-matched controls	144 vs. 48	Chinese	TSH 2.72 ± 1.5 vs. 2.14 ± 0.98 mIU/L *p=*0.003; fT_3_, fT_4_ levels - NS	TSH > 4.78 mIU/L	Anti-TPO positivity 15.28% vs. 6.25% - NS; Anti-Tg positivity 13.2% vs. 12.5% - NS		
2018 (225)	PCOS vs. age-matched healthy women	184 vs. 106	Turkish	TSH and fT_4_ levels - NS	TSH > 4.94 mIU/L	Higher anti-TPO and anti-Tg mean levels *p*<0.001; Anti-TPO positivity 37.9% vs. 11.1% *p*<0.001;Anti-Tg positivity 15.3% vs. 5.1% *p*=0.013		37.9% vs. 11.1% *p*<0.001; Anti-TPO positivity
2018 (226)	PCOS vs. women without PCOS	827 vs. 804	German		All PCOS were euthyroid; TSH >2.5 mIU/L			23% vs. 9.2% *p*<0.05; At least two of three criteria: anti-TPO and/or anti-Tg positivity, TSH levels above the normal range and hypoechoic thyroid
2020 (10)	PCOS vs. age-matched healthy women	210 vs. 343	Korean	TSH and fT_4_ mean levels - NS	TSH > 4.1 mIU/L	Anti-TPO positivity 4.8% vs.7.6% - NS	9.3% vs. 12.3% - NS	PCOS 6.2%; Anti-TPO positivity and/or hypoechoic thyroid
**Study year [ref]**	**Examined group**	***N***	**Population**	**TSH level**		**Prevalence of SCH;TSH cut-off value**		**Metabolic and hormonal parameters**	
2013 (96)	PCOS	168	Brazilian	SCH 6.1 ± 1.2 vs.euthyroidism 2.3 ± 1.0 mIU/L		11.3%; TSH > 4.5 mIU/L		Higher LDL in SCH *p*=0.04	
2014 (227)	PCOS	75	Indian	SCH 6.89 ± 5.52 vs. euthyroidism 1.89 ± 0,78 mIU/L *p*=0.006		25.5%; TSH > 3.75 mIU/L		Lower free testosterone in SCH *p*=0.006	
2014 (5)	PCOS	428	Chinese	SCH 5.94 ± 0.53 mIU/L		14%; TSH > 5 mIU/L		In SCH higher TC *p*=0.049; higher LDL *p*=0.001; lower HDL *p*=0.051; higher dyslipidemia *p*=0.014	
2018 (228)	PCOS	137	U.S.			21.9%; TSH > 2.5 mIU/L		In SCH higher fasting glucose *p*=0.03; higher HOMA-IR *p*=0.03	

AITD, autoimmune thyroid disease; fT_3_, free triiodothyronine; fT_4_, free thyroxine; HDL, high-density lipoprotein cholesterol; HOMA-IR, homeostasis model assessment insulin resistance; LDL, low-density lipoprotein cholesterol; MS, metabolic syndrome; NS, no significant change or similar level; PCOS, polycystic ovary syndrome; SCH, subclinical hypothyroidism; TC, total cholesterol; Tg, thyroglobulin; TPO, thyroid peroxidase; TSH, thyroid-stimulating hormone.

In women with PCOS, compared with those without the condition, higher TSH levels, anti-thyroid antibody positivity rate, and prevalence of thyroid disorders, particularly HT, have been demonstrated in three independent meta-analyses conducted to date (9, 229, 230). Based on six studies, the combined OR of SCH risk for women with PCOS (compared with healthy women) was 2.87 (95% confidence interval (CI), 1.82–9.92; *p* < 10^-6^), assuming a TSH cut-off level of > 2.5 mIU/L, and 3.59 (95% CI, 2.25–5.73; *p* < 10^-6^) when limiting TSH to ≥ 4 mIU/L (229). Similarly, two meta-analyses indicated a significant association between PCOS and the presence of AITD: one included six studies (OR = 4.81; 95% CI, 2.88–8.04; *p* < 10^-5^) (9) and the other included 13 studies (OR = 3.27; 95% CI, 2.32–4.63; *p* < 10^-4^) (230). The higher risk of AITD among women with PCOS also persisted after geographical stratification of the study populations (230).

### Possible Cross-Connections Predisposing to Joint Occurrence of Polycystic Ovary Syndrome and Autoimmune Thyroid Disease

Although the association between PCOS and AITD is generally uncontested, its cause remains unclear. Both syndromes share a number of common clinical and pathological features; however, whether mutual interrelationships are present, or whether one condition predisposes an individual to another disorder, remains speculative (8). Some evidence suggests that PCOS may have an autoimmune background. Abnormally elevated levels of systemic autoimmune markers such as anti-histone, anti-double stranded DNA (anti-dsDNA), and anti-nuclear antibodies, which are considered classic features of autoimmune disease, have been observed in women with PCOS (231, 232); however, the presence of anti-ovarian antibodies in PCOS remains controversial (231).

The most obvious association between PCOS and HT is the increased metabolic risk of obesity, IR, and dyslipidemia (27, 233). Overweight and obese patients with PCOS showed a higher tendency toward thyroid dysfunction; TSH levels > 2.5 mIU/L were significantly more common in patients with BMI > 25 kg/m^2^ (56%) than in those with BMI ≤ 25 (25.8%, *p* < 0.005) (7). Patients with PCOS and AITD were more obese by an average of 2 kg/m² (226). Higher TSH levels (as well as a higher frequency of nodular goiter and thyroid volume) were observed in patients with PCOS, and these parameters correlated with IR (7, 223). Furthermore, fasting glucose and homeostasis model assessment (HOMA)-IR levels among patients with PCOS and SCH were higher than those in euthyroid PCOS patients, independent of BMI (10, 228, 234). Notably, ethnic diversity among euthyroid PCOS patients and patients with PCOS and SCH was suggested with respect to IR and lipid profiles (227). By contrast, SCH is not an independent risk factor for PCOS among obese women of reproductive age (235).

In particular, combined occurrence of PCOS and SCH increases the risk of impaired lipid profiles. Compared with euthyroid PCOS patients, PCOS patients with elevated TSH levels show a trend toward higher triglyceride and LDL cholesterol levels, as well as lower high-density lipoprotein (HDL) cholesterol levels (5, 96, 220). A positive correlation was found between TSH and LDL cholesterol levels, with the optimal TSH cut-off point for elevated LDL cholesterol risk defined as 4.07 mIU/L (5). Furthermore, two recent meta-analyses covering 12 and nine studies demonstrate that the presence of SCH in women with PCOS is associated with an increase in metabolic disorders, particularly dyslipidemia, which affect triglyceride, LDL, HDL, and total cholesterol levels (234). Taken together, current data suggest that the combined effect of PCOS and HT is associated with a higher risk of more pronounced metabolic disorders than either of these syndromes alone.

It has been hypothesized that increased IR in obesity and secretion of pro-inflammatory mediators can lead to elevated TSH levels through one of two pathways: decreased deiodinase-2 activity or increased levels of leptin hormone, which act directly to stimulate increased TRH secretion by the hypothalamus (110, 236). In addition, increased leptin, as a result of weight gain, can mediate autoimmunity by preferential up-regulation of autoreactive T cells and down-regulation of Treg cells, mediating a suppressive effect on the immune system (8). It is suggested that PCOS exacerbates development of SCH, likely *via* obesity and IR (237). Moreover, hypothyroidism may aggravate IR.

One possible explanation for the high prevalence of HT in PCOS assumes that changes in the fetal thymus, and resulting alterations in immune tolerance, may predispose to combined PCOS and HT in adulthood (27). Estrogen or adrenal steroids, such as corticosterone, injected into female mice early in life (before the final stage of thymus development) results in anovulation and follicular cyst formation (238). It is also suggested that estrogen can damage the thymus during its development, and that the resulting absence of Treg cells is a prerequisite for formation of ovarian cysts (238). Similar to PCOS, hypothyroidism can result in ovarian cyst formation (8) and, notably, they are normalized in response to T_4_ replacement (106). CD4^+^ CD25^+^ FOXP3^+^ Treg cells protected against autoimmunity in a murine model of HT (239). The transcription factor, FOXP3, which is involved in Treg cell formation and consequently *FOXP3* expression, is dependent on production of the cytokine TGF-β (239). Since lower levels of TGF-β1 are reported in HT than in healthy women (240), lower levels of Treg cells are also expected. TGF-β1 inhibits proliferation, differentiation, and apoptosis of T cells, as well as increases the growth of naive T cells (241); hence, it is thought to be involved in development of autoimmunity. Furthermore, TGF-β signaling appears to be important for the fetal origin of PCOS and folliculogenesis (242).

The impact of sex hormones on development of autoimmunity appears obvious given the clear predominance of women among patients affected by autoimmune diseases; the ratio of women to men is 15:1 in the case of AITD (243). Estrogen can stimulate the immune system and increase Treg cell formation (244). In fertile, non-pregnant women, the number of CD4^+^ CD25^+^ FOXP3^+^ Treg cells increases in the late follicular phase of the menstrual cycle and decreases in the luteal phase, which correlates with E_2_ levels (245). Unlike estrogen, progesterone levels correlate inversely with dendritic cell secretion of pro-inflammatory cytokine IL6, which in turn inhibits *FOXP3* expression and Treg cell generation (246, 247). Furthermore, progesterone may suppress CD4^+^ T cell proliferation and Th1 responses (248). In women with normal menstrual periods, the immunosuppressive action of progesterone and androgens counteracts the stimulating effect of estrogen (248, 249), while in women with PCOS, a decrease in progesterone levels is usually detected due to irregular menses and oligo- or anovulatory cycles (250). Also, a significantly higher E_2_ level and estrogen-to-progesterone ratio were observed in anti-TPO-positive compared with anti-TPO-negative women with PCOS (219). The resulting imbalance between estrogen and progesterone is thought to be associated with an excessive inflammatory response that promotes autoimmune disorders in PCOS (8). Consistent with this assumption, patients with PCOS exhibit low-grade inflammation, characterized by elevated levels of CRP and independent of obesity (251). PCOS is characterized by an excess of androgens; however, it is speculated that the suppressive effect of androgens on the immune system at the levels observed in PCOS is probably insufficient to prevent autoimmunity (27). In addition, lower testosterone levels, a lower free androgen index, and less severe hyperandrogenemia are observed in patients with PCOS and HT relative to those with PCOS alone (226), although, the results have not been confirmed in patients with PCOS and SCH (234, 252).

Low levels of vitamin D are associated with both PCOS and HT (253, 254) and provide another possible link between these syndromes and autoimmunity. Vitamin D is considered to be protective against autoimmune diseases (255). A strong association has been reported between the severity of vitamin D deficiency and HT, as well as anti-thyroid antibody and thyroid hormone levels (256). Nevertheless, concerns remain that the effects of vitamin D may not be direct, but rather secondary to estrogen dysregulation (257). Consistent with this hypothesis, low levels of 25 hydroxyvitamin D_3_ (25(OH)D_3_) are associated with AITD in pre-menopausal, but not post-menopausal, women (258). A genetic variant of *CYP27B1* hydroxylase, which is responsible for production of an active form of vitamin D from 25(OH)D_3_, is associated with HT (259). Furthermore, polymorphisms in the *VDR* gene causing a decrease in vitamin D levels are associated with both HT and several metabolic syndrome features in women with PCOS (27). Moreover, levels of 25(OH)D_3_ are significantly lower in women with PCOS and AITD than those without AITD (253). Among overweight and obese persons, a significantly higher frequency of vitamin D deficiency was observed in patients with HT than without HT (69% vs. 52%, *p* = 0.042) (260), suggesting that low vitamin D level is not merely a marker of obesity.

### Genetic Predisposition to Combined Polycystic Ovary Syndrome and Hashimoto’s Thyroiditis

As stated above, a strong genetic impact on inheritance and susceptibility to disease development is present in both PCOS and HT. Although a functional relevance of several candidate gene polymorphisms has been suggested, no common genetic background has been established. To date, only a few genetic variants have been proposed to be causally associated with the joint incidence of both disorders. The most convincingly described are three genetic polymorphisms that can contribute to both PCOS and HT. These are polymorphisms in *FBN3*, a gene related to TGF-β activity and Treg cell levels; *CYP1B1*, a gene involved in E_2_ metabolism; and *GNRHR*. In addition, there are two susceptibility loci identified in GWAS that are common for both diseases (**Tables 1** and **2**): *FSHR* (2p16.3) and *INSR* (19p13.3); however, no functional overlap between the two diseases has yet been confirmed.

The *CYP1B1* gene encodes an enzyme belonging to the multi-gene-encoded cytochrome P450 enzyme family, which oxidizes E_2_ to 4-hydroxyestradiol (261). *CYP1B1* is expressed at a level 3 times lower in PCOS ovaries than in control ovaries (262). The pathogenic polymorphism, L432V (rs1056836), in *CYP1B1* is associated with serum T_4_, fT_4_, and fT_3_ concentrations among patients with PCOS (263). Although none of the studied polymorphisms in this gene are associated with disease risk, suggesting that CYP1B1 may not have a causative role in the etiology of PCOS, the *CYP1B1* L432V polymorphism provides a potential link between PCOS and HT.

GnRH is a key hypothalamic peptide that, after binding to a specific receptor, GnRHR, stimulates a release of LH and FSH from pituitary gonadotropic cells. Increased levels of LH and a higher frequency of LH pulses (264), as well as a higher prevalence of increased TSH levels (8), were observed in women with PCOS. GnRH was shown to enhance the release of TSH (265). Furthermore, a 3’-UTR polymorphism in the *GNRHR* gene, rs1038426, was found to affect *GNRHR* expression, with a variant allele-dose effect, and was associated with the concentration of serum TSH as well as insulin secretion and insulin sensitivity in women with PCOS (266). An association between TSH and fasting insulin levels and insulin sensitivity was also reported (267). While *GNRHR* polymorphism did not contribute to the risk of PCOS (266), it links gonadotropin action-related dysfunction with IR and possibly also with thyroid function disorders in PCOS. Several PCOS risk variants were identified in the *INSR* gene, although replication studies produced mostly inconsistent results. The most convincing seems to be an association of rs2252673 in intron 11, identified by the entire gene examination with a tagging approach (268), and silent SNP rs1799817 at exon 17, encoding the tyrosine kinase domain of the INSR (80); especially among lean women with PCOS. The intronic *INSR* variants were also independently identified by the GWAS for PCOS and for the level of TSH (**Tables 1** and **2**); however, the causal variant of this loci and the impact on *INSR* expression remained unknown. Nevertheless, pronounced IR and defects in insulin secretion are commonly associated with PCOS. In women with PCOS, an increase in beta-cell function was observed when compared to the age and BMI matched controls, which was correlated with the intensity of IR (269).

A strong association was observed in candidate gene studies between linked to each other missense *FSHR* gene variants: Thr307Ala (rs6165) and Asn680Ser (rs6166), and intensity of some PCOS clinical traits. The Ser^680^ allele was associated with higher serum gonadotrophic hormones concentrations and higher frequency of hyperandrogenism presence (44). Longer follicular phase and lower E_2_ levels after exogenous FSH stimulation were also observed, suggesting lower sensitivity of this *FSHR* variant (270). Several other *FSHR* polymorphisms have been shown to correlate with ovarian function, including SNPs identified by GWAS in Han Chinese population (**Table 1**) and by fine mapping of 2p16.3 region in a population of European ancestry (271); however, they seem to be associated with FSH levels and the PCOM phenotype rather than with disease risk (33, 47). Recent GWAS among patients with HT identified SNP rs12713034 in the *FSHR* gene that is associated with the presence of anti-thyroid antibodies (146). In addition, hypothyroidism has been found to decrease FSH and E_2_ levels and alter FSHR-mediated expression of *CYP51*, a key enzyme involved in sterols and steroids biosynthesis during folliculogenesis and oocyte maturation, which is regulated by FSH (272); thus providing a further link between the functions of the ovaries and thyroid gland.

FBN3 is a member of the fibrillin/LTBP (latent TGF-β binding protein) family of ubiquitously expressed extracellular matrix proteins that regulate the bioavailability and activity of TGF-β, providing binding sites for its sequestration (273). The *FBN3* genetic variant, D19S884 allele 8 (A8), a dinucleotide repeat microsatellite marker in intron 55, is the variant most strongly associated with PCOS susceptibility at the 19p13.2 locus (274). A rare missense variant (Asp911Val) in *FBN3* was also found by the whole-exome sequencing approach (275). Although the results were not consistent, further studies suggest that D19S884 is likely a causal variant for PCOS susceptibility (275, 276). It was suggested that the A8 allele may affect splicing of *FBN3* transcript (277). Women with PCOS carrying the A8 allele had significantly lower circulating TGF-β1 levels, and higher inhibin B and aldosterone levels, as well as higher levels of fasting INS and HOMA-IR than women with PCOS without the A8 allele (277). It is hypothesized that women with PCOS and the A8 variant, and therefore lower TGF-β levels, are more susceptible to HT than those without this allele (27). Some findings suggest that expression of the *FBN3* gene in fetal ovaries may predispose to PCOS development in later life, supporting the existing hypothesis of the fetal origin of PCOS (278).

As outlined in this review, our current knowledge of the genetic basis of the joint occurrence of PCOS and HT leads us to believe that there may be no strong shared genetic variants associated with the risk of both diseases. Rather, it is a specific combination of risk factors for individual diseases that predisposes them to occur together. Moreover, due to the complex background of both disorders, this combination may be specific not so much to the risk of the disease itself as to the expression of its individual phenotypes. In line with this assumption, it has been recently indicated that the use of combined polygenic and phenotypic risk prediction may improve the accuracy of PCOS diagnosis (279).

**Figure 1:**
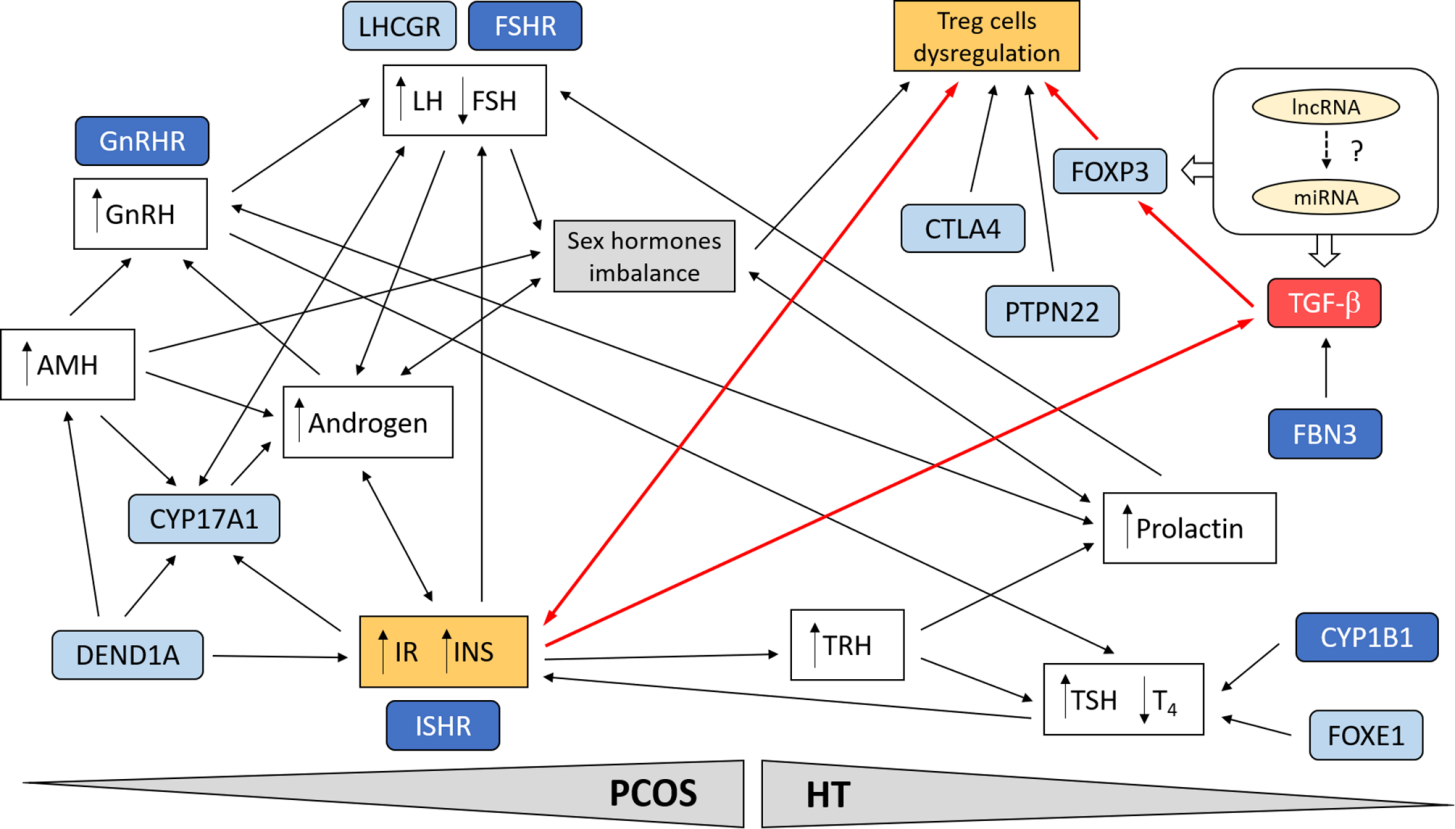
Schematic presentation of possible cross-linkages between PCOS and HT. Involvement of the most important genetic factors and molecular pathways. Treg cell dysregulation emerges as a critical point in the genetic and functional network linking the two diseases. In PCOS, genetic variation emphasizes the contribution of both hormonal imbalance (gonadotropins, androgens, and female sex hormones) and metabolic factors (IR and INS secretion), often interacting through a feedback loop. Suppression of TGF-β signaling pathway in combination with IR may lead to dysregulation of Treg cells and promotion of autoimmunity in women with PCOS (red line connections). Possible interaction of ncRNA (miRNA, lncRNA) with TGF-β signaling. The most important genes are indicated in blue; darker blue designates susceptibility genes for both diseases.

As shown in **Figure 1**, which simplifies the possible cross-linkages between PCOS and HT, the multi-directional link seems to be the best explanation for the predisposition to joint occurrence of both diseases. Treg cells dysregulation emerges as a critical point in the genetic and functional network connecting the two diseases. In PCOS, genetic variation emphasizes the contribution of both hormonal imbalance (gonadotropins, androgens, and female sex hormones) and metabolic factors (IR and INS secretion), often interacting through a feedback loop (230, 248). It can be assumed that the favorable hormonal and metabolic background in women with PCOS may predispose them to thyroid autoimmunity and aggravate the disease symptoms through the HT susceptibility factors (8, 219).

The best documented genetic association between PCOS and HT relates to the TGF-β signaling pathway. Factors involved in this pathway are clearly good candidate susceptibility genes for both syndromes, since they have key roles in the immune system, hormone regulation, inflammation, cell proliferation, tissue differentiation, apoptosis, and related metabolic consequences such as IR. In PCOS, the inflammation of visceral adipose tissue resulting in chronic release of pro-inflammatory cytokines is a major contributor to IR (280). Treg cells suppress pro-inflammatory effects of autoreactive T cells (281). Depletion of CD4^+^CD25^+^Foxp3^+^ Treg cells and increased inflammation in visceral adipose tissues was found to contribute to IR in HT (282). We are hypothesizing that the suppression of TGF-β signaling pathway in combination with IR may lead to Treg cells dysregulation and promotion of autoimmunity in women with PCOS (**Figure 1**). Beside *FBN3*, no members of the TGF-β signaling have been shown to be among the top GWAS associations for PCOS or HT. However, rs4803457 polymorphism in *TGFB1* gene was associated with PCOS susceptibility in candidate gene studies in Chinese Han women (273). The genetic variation in transcription factor *FOXP3* has also been reported in candidate gene studies for AITD (2). FOXP3 is a crucial regulator of Treg cells differentiation and function and its expression is induced by TGF-β, which activity, in turn, is regulated by FBNs (33).

In recent years, there has been an increase in evidence of the role of non-coding RNA (ncRNA) in the development of various diseases. Differentially expressed microRNA (miRNA) and long ncRNA (lncRNA) were identified both in PCOS and HT (283, 284). The ncRNAs were shown to be involved in regulating T cell production and differentiation (285). Furthermore, several ncRNAs were involved in regulation by the TGF-β signaling pathway. In HT, the expression of miR-141 was downregulated due to its involvement in TGF-β pathway and regulation through IL2 (286). In turn, lncRNA-IFNG-AS1 was upregulated in HT patients, and it was associated with IFN-γ expression in human CD4+ T cells and with the frequency of circulating Th1 cells (287). The lncRNA TGFB2-AS1 was shown to be transcriptionally regulated by TGF-β and to suppress TGF-β/BMP-mediated response of target genes (288). In granulosa cells from patients with PCOS, expression of miR-423 was downregulated, and both miR-33b and miR-142 were upregulated, compared to controls; miR-423 directly suppressed SMAD7, while miR-33b and miR-142 targeted TGF-β receptor 1 (TGFBR1) (289). Several lncRNA have been reported to positively regulate TGF-β/SMAD signaling (290). The lncRNAs can act directly or as miRNA sponges, thus reducing their regulatory effect on target mRNAs (291). In granulosa cells, lncRNA-NORFA directly interacts with miR-126 and prevents it from binding to TGFBR2 3’UTR (292). Another lncRNA, MALAT1, has been found to regulate TGF-β signaling through sponging miR-125b and miR-203a, TGF-β negative regulators by targeting TGFBR1 and TGFBR2 (293). Considering the above-mentioned examples of the role of ncRNA in modulating of TGF-β signaling, the involvement of ncRNA in the processes leading to the joint occurrence of PCOS and HT seems very likely; however, there are no strong data supporting this hypothesis and it requires confirmation in further studies.

## Conclusions

PCOS and HT are two of the most common endocrine disorders among young women worldwide. Both syndromes are causally related to the risk of severe metabolic and reproductive disorders and are significant social problems. Thyroid dysfunction in PCOS appears to enhance the clinical symptoms of the disease and the severity of its consequences. The pathogenesis of the association of PCOS with HT is not clear, although the relationship between thyroid hormones and ovary function is indisputable. There is good evidence for a strong genetic impact on the development of both diseases; hence, a common genetic predisposition is possible. However, to date, it is not apparent. Furthermore, the question of whether PCOS predisposes to development HT, or whether HT is a forerunner of PCOS, remains open.

## Author Contributions

All authors contributed to the article and approved the submitted version. NZ-L wrote the original draft. EH wrote and critically revised the manuscript.

## Funding

This work was supported by the Polish National Science Centre (grant number 2016/23/B/NZ2/00696) and the Centre of Postgraduate Medical Education (grant number 501-1009-12-20).

## Conflict of Interest

The authors declare that the research was conducted in the absence of any commercial or financial relationships that could be construed as a potential conflict of interest.

## References

[1] Ben-Shlomo I Younis JS. Basic research in PCOS: are we reaching new frontiers? Reprod BioMed Online (2014) 28:669–83. 10.1016/j.rbmo.2014.02.011 24768413

[2] Lee HJ Li CW Hammerstad SS Stefan M Tomer Y. Immunogenetics of autoimmune thyroid diseases: A comprehensive review. J Autoimmun (2015) 64:82–90. 10.1016/j.jaut.2015.07.009 26235382 PMC4628844

[3] Caturegli P De Remigis A Rose NR. Hashimoto thyroiditis: clinical and diagnostic criteria. Autoimmun Rev (2014) 13:391–7. 10.1016/j.autrev.2014.01.007 24434360

[4] Lim SS Davies MJ Norman RJ Moran LJ. Overweight, obesity and central obesity in women with polycystic ovary syndrome: a systematic review and meta-analysis. Hum Reprod Update (2012) 18:618–37. 10.1093/humupd/dms030 22767467

[5] Huang R Zheng J Li S Tao T Liu W. Subclinical hypothyroidism in patients with polycystic ovary syndrome: distribution and its association with lipid profiles. Eur J Obstet Gynecol Reprod Biol (2014) 177:52–6. 10.1016/j.ejogrb.2014.04.013 24768234

[6] Novais J de SM Benetti-Pinto CL Garmes HM Jales RM Juliato CRT. Polycystic ovary syndrome and chronic autoimmune thyroiditis. Gynecol Endocrinol (2015) 31:48–51. 10.3109/09513590.2014.958990 25211537

[7] Morgante G Musacchio MC Orvieto R Massaro MG De Leo V. Alterations in thyroid function among the different polycystic ovary syndrome phenotypes. Gynecol Endocrinol (2013) 29:967–9. 10.3109/09513590.2013.829445 23957782

[8] Singla R Gupta Y Khemani M Aggarwal S. Thyroid disorders and polycystic ovary syndrome: An emerging relationship. Indian J Endocrinol Metab (2015) 19:25–9. 10.4103/2230-8210.146860 PMC4287775 25593822

[9] Du D Li X. The relationship between thyroiditis and polycystic ovary syndrome: a meta-analysis. Int J Clin Exp Med (2013) 6:880–9. PMC3832324 24260593

[10] Kim JJ Yoon JW Kim MJ Kim SM Hwang KR Choi YM. Thyroid autoimmunity markers in women with polycystic ovary syndrome and controls. Hum Fertil Camb Engl (2020) 7:1–7. 10.1080/14647273.2019.1709668 31910041

[11] Ott J Aust S Kurz C Nouri K Wirth S Huber JC. Elevated antithyroid peroxidase antibodies indicating Hashimoto’s thyroiditis are associated with the treatment response in infertile women with polycystic ovary syndrome. Fertil Steril (2010) 94:2895–7. 10.1016/j.fertnstert.2010.05.063 20638057

[12] Azziz R Carmina E Chen Z Dunaif A Laven JSE Legro RS. Polycystic ovary syndrome. Nat Rev Dis Primers (2016) 2:16057. 10.1038/nrdp.2016.57 27510637

[13] Rotterdam ESHRE/ASRM-Sponsored PCOS Consensus Workshop Group. Revised 2003 consensus on diagnostic criteria and long-term health risks related to polycystic ovary syndrome. Fertil Steril (2004) 81:19–25. 10.1016/j.fertnstert.2003.10.004 14711538

[14] Crespo RP Bachega TASS Mendonça BB Gomes LG. An update of genetic basis of PCOS pathogenesis. Arch Endocrinol Metab (2018) 62:352–61. 10.20945/2359-3997000000049 PMC10118782 29972435

[15] Azziz R. Introduction: Determinants of polycystic ovary syndrome. Fertil Steril (2016) 106:4–5. 10.1016/j.fertnstert.2016.05.009 27238627

[16] McCartney CR Marshall JC. CLINICAL PRACTICE. Polycystic Ovary Syndrome. N Engl J Med (2016) 375:54–64. 10.1056/NEJMcp1514916 27406348 PMC5301909

[17] Li Y Chen C Ma Y Xiao J Luo G Li Y. Multi-system reproductive metabolic disorder: significance for the pathogenesis and therapy of polycystic ovary syndrome (PCOS). Life Sci (2019) 228:167–75. 10.1016/j.lfs.2019.04.046 31029778

[18] Wild RA Rizzo M Clifton S Carmina E. Lipid levels in polycystic ovary syndrome: systematic review and meta-analysis. Fertil Steril (2011) 95:1073–1079.e11. 10.1016/j.fertnstert.2010.12.027 21247558

[19] Song DK Lee H Hong YS Sung Y-A. Insulin resistance is associated with hirsutism in unselected reproductive-aged women. Clin Endocrinol (Oxf) (2019) 90:586–91. 10.1111/cen.13936 30657205

[20] Lim SS Kakoly NS Tan JWJ Fitzgerald G Bahri Khomami M Joham AE. Metabolic syndrome in polycystic ovary syndrome: a systematic review, meta-analysis and meta-regression. Obes Rev (2019) 20:339–52. 10.1111/obr.12762 30339316

[21] Spritzer PM Lecke SB Satler F Morsch DM. Adipose tissue dysfunction, adipokines, and low-grade chronic inflammation in polycystic ovary syndrome. Reproduction (2015) 149:R219–27. 10.1530/REP-14-0435 25628442

[22] Xiong Y Liang X Yang X Li Y Wei L. Low-grade chronic inflammation in the peripheral blood and ovaries of women with polycystic ovarian syndrome. Eur J Obstet Gynecol Reprod Biol (2011) 159:148–50. 10.1016/j.ejogrb.2011.07.012 21908093

[23] Spritzer PM Barone CR Oliveira FB. Hirsutism in Polycystic Ovary Syndrome: Pathophysiology and Management. Curr Pharm Des (2016) 22:5603–13. 10.2174/1381612822666160720151243 27510481

[24] Romanowski MD Parolin MB Freitas ACT Piazza MJ Basso J Urbanetz AA. Prevalence of non-alcoholic fatty liver disease in women with polycystic ovary syndrome and its correlation with metabolic syndrome. Arq Gastroenterol (2015) 52:117–23. 10.1590/S0004-28032015000200008 26039829

[25] Condorelli RA Calogero AE Mauro MD Vignera SL. PCOS and diabetes mellitus: from insulin resistance to altered beta pancreatic function, a link in evolution. Gynecol Endocrinol (2017) 33:665–7. 10.1080/09513590.2017.1342240 28644709

[26] Gunning MN Fauser BCJM. Are women with polycystic ovary syndrome at increased cardiovascular disease risk later in life? Climacteric (2017) 20:222–7. 10.1080/13697137.2017.1316256 28457146

[27] Gaberšček S Zaletel K Schwetz V Pieber T Obermayer-Pietsch B Lerchbaum E. Mechanisms in endocrinology: thyroid and polycystic ovary syndrome. Eur J Endocrinol (2015) 172:R9–21. 10.1530/EJE-14-0295 25422352

[28] Kerchner A Lester W Stuart SP Dokras A. Risk of depression and other mental health disorders in women with polycystic ovary syndrome: a longitudinal study. Fertil Steril (2009) 91:207–12. 10.1016/j.fertnstert.2007.11.022 18249398

[29] Abbott DH Dumesic DA Levine JE. Hyperandrogenic origins of polycystic ovary syndrome - implications for pathophysiology and therapy. Expert Rev Endocrinol Metab (2019) 14:131–43. 10.1080/17446651.2019.1576522 PMC6992448 30767580

[30] Reid SP Kao C-N Pasch L Shinkai K Cedars MI Huddleston HG. Ovarian morphology is associated with insulin resistance in women with polycystic ovary syndrome: a cross sectional study. Fertil Res Pract (2017) 3:8. 10.1186/s40738-017-0035-z 28620546 PMC5450099

[31] Cadagan D Khan R Amer S. Thecal cell sensitivity to luteinizing hormone and insulin in polycystic ovarian syndrome. Reprod Biol (2016) 16:53–60. 10.1016/j.repbio.2015.12.006 26952754

[32] Vink JM Sadrzadeh S Lambalk CB Boomsma DI. Heritability of polycystic ovary syndrome in a Dutch twin-family study. J Clin Endocrinol Metab (2006) 91:2100–4. 10.1210/jc.2005-1494 16219714

[33] Jones MR Goodarzi MO. Genetic determinants of polycystic ovary syndrome: progress and future directions. Fertil Steril (2016) 106:25–32. 10.1016/j.fertnstert.2016.04.040 27179787

[34] Deswal R Yadav A Dang AS. Sex hormone binding globulin - an important biomarker for predicting PCOS risk: A systematic review and meta-analysis. Syst Biol Reprod Med (2018) 64:12–24. 10.1080/19396368.2017.1410591 29227165

[35] Patel S. Polycystic ovary syndrome (PCOS), an inflammatory, systemic, lifestyle endocrinopathy. J Steroid Biochem Mol Biol (2018) 182:27–36. 10.1016/j.jsbmb.2018.04.008 29678491

[36] Hiam D Moreno-Asso A Teede HJ Laven JSE Stepto NK Moran LJ. The Genetics of Polycystic Ovary Syndrome: An Overview of Candidate Gene Systematic Reviews and Genome-Wide Association Studies. J Clin Med (2019) 8:1606. 10.3390/jcm8101606 PMC6832583 31623391

[37] Zhao H Lv Y Li L Chen Z-J. Genetic Studies on Polycystic Ovary Syndrome. Best Pract Res Clin Obstet Gynaecol (2016) 37:56–65. 10.1016/j.bpobgyn.2016.04.002 27264388

[38] Chen D-J Ding R Cao J-Y Zhai J-X Zhang J-X Ye D-Q. Two follicle-stimulating hormone receptor polymorphisms and polycystic ovary syndrome risk: a meta-analysis. Eur J Obstet Gynecol Reprod Biol (2014) 182:27–32. 10.1016/j.ejogrb.2014.08.014 25218548

[39] Liu XB Deng XH Zhou B Zhang L Niu XM. Meta-analysis of the correlation between the TNF-α308G/A polymorphism and polycystic ovary syndrome. Genet Mol Res (2016) 15:1–9. 10.4238/gmr.15027923 27323168

[40] Shi X Xie X Jia Y Li S. Associations of insulin receptor and insulin receptor substrates genetic polymorphisms with polycystic ovary syndrome: A systematic review and meta-analysis. J Obstet Gynaecol Res (2016) 42:844–54. 10.1111/jog.13002 27098445

[41] Liu AL Xie HJ Xie HY Liu J Yin J Hu JS. Association between fat mass and obesity associated (FTO) gene rs9939609 A/T polymorphism and polycystic ovary syndrome: a systematic review and meta-analysis. BMC Med Genet (2017) 18:89. 10.1186/s12881-017-0452-1 28826396 PMC5563909

[42] Liao D Yu H Han L Zhong C Ran X Wang D. Association of PON1 gene polymorphisms with polycystic ovarian syndrome risk: a meta-analysis of case–control studies. J Endocrinol Invest (2018) 41(11):1289–300. 10.1007/s40618-018-0866-4 29546656

[43] Li Y Zhu H Liu M Zeng Z Zeng Y Xu X. Significant association between methylenetetrahydrofolate reductase gene C677T polymorphism with polycystic ovary syndrome risk: A meta-analysis update. Medicine (Baltimore) (2020) 99(4):e18720. 10.1097/MD.0000000000018720 31977861 PMC7004748

[44] Valkenburg O Uitterlinden AG Piersma D Hofman A Themmen APN de Jong FH. Genetic polymorphisms of GnRH and gonadotrophic hormone receptors affect the phenotype of polycystic ovary syndrome. Hum Reprod Oxf Engl (2009) 24:2014–22. 10.1093/humrep/dep113 19403562

[45] Wang F Niu W-B Kong H-J Guo Y-H Sun Y-P. The role of AMH and its receptor SNP in the pathogenesis of PCOS. Mol Cell Endocrinol (2017) 439:363–8. 10.1016/j.mce.2016.09.023 27664518

[46] Chen L Zhang Z Huang J Jin M. Association between rs1800795 polymorphism in the interleukin-6 gene and the risk of polycystic ovary syndrome. Medicine (Baltimore) (2018) 97:e11558. 10.1097/MD.0000000000011558 30024552 PMC6086475

[47] Laven JSE. Follicle Stimulating Hormone Receptor (FSHR) Polymorphisms and Polycystic Ovary Syndrome (PCOS). Front Endocrinol (2019) 10:23. 10.3389/fendo.2019.00023 PMC6379247 30809190

[48] Shi X-Y Huang A-P Xie D-W Yu X-L. Association of vitamin D receptor gene variants with polycystic ovary syndrome: a meta-analysis. BMC Med Genet (2019) 20:32. 10.1186/s12881-019-0763-5 30764792 PMC6376757

[49] Wu H Yu K Yang Z. Associations between TNF-α and interleukin gene polymorphisms with polycystic ovary syndrome risk: a systematic review and meta-analysis. J Assist Reprod Genet (2015) 32:625–34. 10.1007/s10815-015-0449-7 PMC4380887 25690158

[50] Lee H Oh J-Y Sung Y-A Chung H Kim H-L Kim GS. Genome-wide association study identified new susceptibility loci for polycystic ovary syndrome. Hum Reprod (2015) 30:723–31. 10.1093/humrep/deu352 25574032

[51] Chen Z-J Zhao H He L Shi Y Qin Y Shi Y. Genome-wide association study identifies susceptibility loci for polycystic ovary syndrome on chromosome 2p16.3, 2p21 and 9q33.3. Nat Genet (2011) 43:55–9. 10.1038/ng.732 21151128

[52] Shi Y Zhao H Shi Y Cao Y Yang D Li Z. Genome-wide association study identifies eight new risk loci for polycystic ovary syndrome. Nat Genet (2012) 44:1020–5. 10.1038/ng.2384 22885925

[53] Hwang J-Y Lee E-J Jin Go M Sung Y-A Lee HJ Heon Kwak S. Genome-wide association study identifies *GYS2* as a novel genetic factor for polycystic ovary syndrome through obesity-related condition. J Hum Genet (2012) 57:660–4. 10.1038/jhg.2012.92 22951595

[54] Hayes MG Urbanek M Ehrmann DA Armstrong LL Lee JY Sisk R. Genome-wide association of polycystic ovary syndrome implicates alterations in gonadotropin secretion in European ancestry populations. Nat Commun (2015) 6:7502. 10.1038/ncomms8502 26284813 PMC4557132

[55] Louwers YV Stolk L Uitterlinden AG Laven JSE. Cross-ethnic meta-analysis of genetic variants for polycystic ovary syndrome. J Clin Endocrinol Metab (2013) 98:E2006–2012. 10.1210/jc.2013-2495 24106282

[56] Ha L Shi Y Zhao J Li T Chen Z-J. Association Study between Polycystic Ovarian Syndrome and the Susceptibility Genes Polymorphisms in Hui Chinese Women. PLoS One (2015) 10:e0126505. 10.1371/journal.pone.0126505 25978310 PMC4433204

[57] Xia J-Y Tian W Yin G-H Yan H. Association of Rs13405728, Rs12478601, and Rs2479106 single nucleotide polymorphisms and in vitro fertilization and embryo transfer efficacy in patients with polycystic ovarian syndrome: A case control genome-wide association study. Kaohsiung J Med Sci (2019) 35:49–55. 10.1002/kjm2.12008 30844144 PMC11900693

[58] Xu Y Li Z Ai F Chen J Xing Q Zhou P. Systematic Evaluation of Genetic Variants for Polycystic Ovary Syndrome in a Chinese Population. PLoS One (2015) 10:e0140695. 10.1371/journal.pone.0140695 26474478 PMC4608705

[59] Day FR Hinds DA Tung JY Stolk L Styrkarsdottir U Saxena R. Causal mechanisms and balancing selection inferred from genetic associations with polycystic ovary syndrome. Nat Commun (2015) 6:8464. 10.1038/ncomms9464 26416764 PMC4598835

[60] Welt CK Styrkarsdottir U Ehrmann DA Thorleifsson G Arason G Gudmundsson JA. Variants in DENND1A are associated with polycystic ovary syndrome in women of European ancestry. J Clin Endocrinol Metab (2012) 97:E1342–1347. 10.1210/jc.2011-3478 PMC3387396 22547425

[61] Lerchbaum E Trummer O Giuliani A Gruber H-J Pieber TR Obermayer-Pietsch B. Susceptibility loci for polycystic ovary syndrome on chromosome 2p16.3, 2p21, and 9q33.3 in a cohort of Caucasian women. Horm Metab Res (2011) 43:743–7. 10.1055/s-0031-1286279 22009367

[62] Eriksen MB Brusgaard K Andersen M Tan Q Altinok ML Gaster M. Association of polycystic ovary syndrome susceptibility single nucleotide polymorphism rs2479106 and PCOS in Caucasian patients with PCOS or hirsutism as referral diagnosis. Eur J Obstet Gynecol Reprod Biol (2012) 163:39–42. 10.1016/j.ejogrb.2012.03.020 22504079

[63] Goodarzi MO Jones MR Li X Chua AK Garcia OA Chen Y-DI. Replication of association of DENND1A and THADA variants with polycystic ovary syndrome in European cohorts. J Med Genet (2012) 49:90–5. 10.1136/jmedgenet-2011-100427 PMC3536488 22180642

[64] Brower MA Jones MR Rotter JI Krauss RM Legro RS Azziz R. Further investigation in europeans of susceptibility variants for polycystic ovary syndrome discovered in genome-wide association studies of Chinese individuals. J Clin Endocrinol Metab (2015) 100:E182–186. 10.1210/jc.2014-2689 PMC4283012 25303487

[65] Zhao S Tian Y Gao X Zhang X Liu H You L. Family-based analysis of eight susceptibility loci in polycystic ovary syndrome. Sci Rep (2015) 5:12619. 10.1038/srep12619 26220222 PMC4518258

[66] Day F Karaderi T Jones MR Meun C He C Drong A. Large-scale genome-wide meta-analysis of polycystic ovary syndrome suggests shared genetic architecture for different diagnosis criteria. PLoS Genet (2018) 14:e1007813. 10.1371/journal.pgen.1007813 30566500 PMC6300389

[67] Gao J Xue J-D Li Z-C Zhou L Chen C. The association of DENND1A gene polymorphisms and polycystic ovary syndrome risk: a systematic review and meta-analysis. Arch Gynecol Obstet (2016) 294:1073–80. 10.1007/s00404-016-4159-x 27488699

[68] Dallel M Sarray S Douma Z Hachani F Al-Ansari AK Letaifa DB. Differential association of DENND1A genetic variants with polycystic ovary syndrome in Tunisian but not Bahraini Arab women. Gene (2018) 647:79–84. 10.1016/j.gene.2018.01.028 29325736

[69] Gammoh E Arekat MR Saldhana FL Madan S Ebrahim BH Almawi WY. DENND1A gene variants in Bahraini Arab women with polycystic ovary syndrome. Gene (2015) 560:30–3. 10.1016/j.gene.2015.01.034 25626177

[70] Bao S Cai JH Yang SY Ren Y Feng T Jin T. Association of DENND1A Gene Polymorphisms with Polycystic Ovary Syndrome: A Meta-Analysis. J Clin Res Pediatr Endocrinol (2016) 8:135–43. 10.4274/jcrpe.2259 PMC5096467 26757598

[71] Tian Y Zhao H Chen H Peng Y Cui L Du Y. Variants in FSHB Are Associated With Polycystic Ovary Syndrome and Luteinizing Hormone Level in Han Chinese Women. J Clin Endocrinol Metab (2016) 101:2178–84. 10.1210/jc.2015-3776 26938199

[72] Venables JP Dalgliesh C Paronetto MP Skitt L Thornton JK Saunders PT. SIAH1 targets the alternative splicing factor T-STAR for degradation by the proteasome. Hum Mol Genet (2004) 13:1525–34. 10.1093/hmg/ddh165 15163637

[73] Lovelace DL Gao Z Mutoji K Song YC Ruan J Hermann BP. The regulatory repertoire of PLZF and SALL4 in undifferentiated spermatogonia. Development Camb Engl (2016) 143:1893–906. 10.1242/dev.132761 PMC4920160 27068105

[74] Kommagani R Szwarc MM Vasquez YM Peavey MC Mazur EC Gibbons WE. The Promyelocytic Leukemia Zinc Finger Transcription Factor Is Critical for Human Endometrial Stromal Cell Decidualization. PLoS Genet (2016) 12:e1005937. 10.1371/journal.pgen.1005937 27035670 PMC4817989

[75] Shim U Kim H-N Lee H Oh J-Y Sung Y-A Kim H-L. Pathway Analysis Based on a Genome-Wide Association Study of Polycystic Ovary Syndrome. PLoS One (2015) 10:e0136609. 10.1371/journal.pone.0136609 26308735 PMC4550465

[76] Pau CT Mosbruger T Saxena R Welt CK. Phenotype and Tissue Expression as a Function of Genetic Risk in Polycystic Ovary Syndrome. PLoS One (2017) 12:e0168870. 10.1371/journal.pone.0168870 28068351 PMC5221814

[77] Jones MR Brower MA Xu N Cui J Mengesha E Chen Y-DI. Systems Genetics Reveals the Functional Context of PCOS Loci and Identifies Genetic and Molecular Mechanisms of Disease Heterogeneity. PLoS Genet (2015) 11:e1005455. 10.1371/journal.pgen.1005455 26305227 PMC4549292

[78] Zhu K Li S Liu J Hong Y Chen Z-J Du Y. Role of RAB5A in FSHR-mediated signal transduction in human granulosa cells. Reproduction Camb Engl (2018) 155:505–14. 10.1530/REP-18-0015 29626103

[79] Li M Zhao H Zhao S-G Wei D-M Zhao Y-R Huang T. The HMGA2-IMP2 Pathway Promotes Granulosa Cell Proliferation in Polycystic Ovary Syndrome. J Clin Endocrinol Metab (2019) 104:1049–59. 10.1210/jc.2018-00544 PMC6753588 30247605

[80] McAllister JM Legro RS Modi BP Strauss JF. Functional genomics of PCOS: from GWAS to molecular mechanisms. Trends Endocrinol Metab (2015) 26:118–24. 10.1016/j.tem.2014.12.004 PMC4346470 25600292

[81] McAllister JM Modi B Miller BA Biegler J Bruggeman R Legro RS. Overexpression of a DENND1A isoform produces a polycystic ovary syndrome theca phenotype. Proc Natl Acad Sci U S A (2014) 111:E1519–1527. 10.1073/pnas.1400574111 PMC3992676 24706793

[82] Kulkarni R Teves ME Han AX McAllister JM Strauss JF. Colocalization of Polycystic Ovary Syndrome Candidate Gene Products in Theca Cells Suggests Novel Signaling Pathways. J Endocr Soc (2019) 3:2204–23. 10.1210/js.2019-00169 PMC6839531 31723719

[83] McAllister JM Han AX Modi BP Teves ME Mavodza GR Anderson ZL. miRNA Profiling Reveals miRNA-130b-3p Mediates DENND1A Variant 2 Expression and Androgen Biosynthesis. Endocrinology (2019) 160:1964–81. 10.1210/en.2019-00013 PMC6656421 31184707

[84] Gorsic LK Kosova G Werstein B Sisk R Legro RS Hayes MG. Pathogenic Anti-Müllerian Hormone Variants in Polycystic Ovary Syndrome. J Clin Endocrinol Metab (2017) 102:2862–72. 10.1210/jc.2017-00612 PMC5546867 28505284

[85] Caburet S Fruchter RB Legois B Fellous M Shalev S Veitia RA. A homozygous mutation of GNRHR in a familial case diagnosed with polycystic ovary syndrome. Eur J Endocrinol (2017) 176:K9–K14. 10.1530/EJE-16-0968 28348023

[86] Bedecarrats GY Linher KD Kaiser UB. Two Common Naturally Occurring Mutations in the Human Gonadotropin-Releasing Hormone (GnRH) Receptor Have Differential Effects on Gonadotropin Gene Expression and on GnRH-Mediated Signal Transduction. J Clin Endocrinol Metab (2003) 88:834–43. 10.1210/jc.2002-020806 12574221

[87] Dapas M Sisk R Legro RS Urbanek M Dunaif A Hayes MG. Family-Based Quantitative Trait Meta-Analysis Implicates Rare Noncoding Variants in DENND1A in Polycystic Ovary Syndrome. J Clin Endocrinol Metab (2019) 104:3835–50. 10.1210/jc.2018-02496 PMC6660913 31038695

[88] McLeod DSA Cooper DS. The incidence and prevalence of thyroid autoimmunity. Endocrine (2012) 42:252–65. 10.1007/s12020-012-9703-2 22644837

[89] Antonelli A Ferrari SM Corrado A Di Domenicantonio A Fallahi P. Autoimmune thyroid disorders. Autoimmun Rev (2015) 14:174–80. 10.1016/j.autrev.2014.10.016 25461470

[90] Hollowell JG Staehling NW Flanders WD Hannon WH Gunter EW Spencer CA. Serum TSH, T(4), and thyroid antibodies in the United States population (1988 to 1994): National Health and Nutrition Examination Survey (NHANES III). J Clin Endocrinol Metab (2002) 87:489–99. 10.1210/jcem.87.2.8182 11836274

[91] Chardès T Chapal N Bresson D Bès C Giudicelli V Lefranc M-P. The human anti-thyroid peroxidase autoantibody repertoire in Graves’ and Hashimoto’s autoimmune thyroid diseases. Immunogenetics (2002) 54:141–57. 10.1007/s00251-002-0453-9 12073143

[92] Fröhlich E Wahl R. Thyroid Autoimmunity: Role of Anti-thyroid Antibodies in Thyroid and Extra-Thyroidal Diseases. Front Immunol (2017) 8:521. 10.3389/fimmu.2017.00521 28536577 PMC5422478

[93] Eschler DC Hasham A Tomer Y. Cutting edge: the etiology of autoimmune thyroid diseases. Clin Rev Allergy Immunol (2011) 41:190–7. 10.1007/s12016-010-8245-8 PMC3129418 21234711

[94] Ajjan RA Weetman AP. The Pathogenesis of Hashimoto’s Thyroiditis: Further Developments in our Understanding. Horm Metab Res (2015) 47:702–10. 10.1055/s-0035-1548832 26361257

[95] Erdogan M Kulaksizoglu M Ganidagli S Berdeli A. Fas/FasL gene polymorphism in patients with Hashimoto’s thyroiditis in Turkish population. J Endocrinol Invest (2017) 40:77–82. 10.1007/s40618-016-0534-5 27572459

[96] Benetti-Pinto CL Piccolo VRSB Garmes HM Juliato CRT. Subclinical hypothyroidism in young women with polycystic ovary syndrome: an analysis of clinical, hormonal, and metabolic parameters. Fertil Steril (2013) 99:588–92. 10.1016/j.fertnstert.2012.10.006 23103018

[97] Canaris GJ Manowitz NR Mayor G Ridgway EC. The Colorado thyroid disease prevalence study. Arch Intern Med (2000) 160:526–34. 10.1001/archinte.160.4.526 10695693

[98] Radetti G. Clinical aspects of Hashimoto’s thyroiditis. Endocr Dev (2014) 26:158–70. 10.1159/000363162 25231451

[99] Krassas GE Poppe K Glinoer D. Thyroid function and human reproductive health. Endocr Rev (2010) 31:702–55. 10.1210/er.2009-0041 20573783

[100] Verma I Sood R Juneja S Kaur S. Prevalence of hypothyroidism in infertile women and evaluation of response of treatment for hypothyroidism on infertility. Int J Appl Basic Med Res (2012) 2:17–9. 10.4103/2229-516X.96795 PMC3657979 23776802

[101] Moleti M Sturniolo G Mauro MD Russo M Vermiglio F. Autoimmune thyroid diseases and pregnancy. Ann Thyroid (2018) 3:1–12. 10.21037/aot.2018.07.03

[102] Zhang Y Wang H Pan X Teng W Shan Z. Patients with subclinical hypothyroidism before 20 weeks of pregnancy have a higher risk of miscarriage: A systematic review and meta-analysis. PLoS One (2017) 12:e0175708. 10.1371/journal.pone.0175708 28414788 PMC5393567

[103] Saran S Gupta BS Philip R Singh KS Bende SA Agroiya P. Effect of hypothyroidism on female reproductive hormones. Indian J Endocrinol Metab (2016) 20:108–13. 10.4103/2230-8210.172245 PMC4743370 26904478

[104] Goel P Kahkasha Narang S Gupta BK Goel K. Evaluation of Serum Prolactin Level in Patients of Subclinical and Overt Hypothyroidism. J Clin Diagn Res (2015) 9:BC15–7. 10.7860/JCDR/2015/9982.5443 PMC4347066 25737975

[105] Dharmshaktu P Kutiyal A Dhanwal D. Vanishing large ovarian cyst with thyroxine therapy. Endocrinol Diabetes Metab Case Rep (2013) 2013:130050. 10.1530/EDM-13-0050 24683475 PMC3965274

[106] Muderris II Boztosun A Oner G Bayram F. Effect of thyroid hormone replacement therapy on ovarian volume and androgen hormones in patients with untreated primary hypothyroidism. Ann Saudi Med (2011) 31:145–51. 10.4103/0256-4947.77500 PMC3102473 21403408

[107] Maratou E Hadjidakis DJ Kollias A Tsegka K Peppa M Alevizaki M. Studies of insulin resistance in patients with clinical and subclinical hypothyroidism. Eur J Endocrinol (2009) 160:785–90. 10.1530/EJE-08-0797 19141606

[108] Asvold BO Bjøro T Nilsen TIL Vatten LJ. Association between blood pressure and serum thyroid-stimulating hormone concentration within the reference range: a population-based study. J Clin Endocrinol Metab (2007) 92:841–5. 10.1210/jc.2006-2208 17200168

[109] Pearce EN. Update in lipid alterations in subclinical hypothyroidism. J Clin Endocrinol Metab (2012) 97:326–33. 10.1210/jc.2011-2532 22205712

[110] Duntas LH Biondi B. The interconnections between obesity, thyroid function, and autoimmunity: the multifold role of leptin. Thyroid (2013) 23:646–53. 10.1089/thy.2011.0499 22934923

[111] Ong KK Kuh D Pierce M Franklyn JA. Childhood Weight Gain and Thyroid Autoimmunity at Age 60–64 Years: The 1946 British Birth Cohort Study. J Clin Endocrinol Metab (2013) 98:1435–42. 10.1210/jc.2012-3761 PMC3651609 23436917

[112] Hansen PS Brix TH Bennedbæk FN Bonnema SJ Iachine I Kyvik KO. The relative importance of genetic and environmental factors in the aetiology of thyroid nodularity: a study of healthy Danish twins. Clin Endocrinol (Oxf) (2005) 62:380–6. 10.1111/j.1365-2265.2005.02230.x 15730424

[113] Brix TH Kyvik KO Hegedüs L. A population-based study of chronic autoimmune hypothyroidism in Danish twins. J Clin Endocrinol Metab (2000) 85:536–9. 10.1210/jcem.85.2.6385 10690851

[114] Zaletel K Gaberšček S. Hashimoto’s Thyroiditis: From Genes to the Disease. Curr Genomics (2011) 12:576–88. 10.2174/138920211798120763 PMC3271310 22654557

[115] Xiao L Muhali F-S Cai T Song R Hu R Shi X. Association of single-nucleotide polymorphisms in the STAT3 gene with autoimmune thyroid disease in Chinese individuals. Funct Integr Genomics (2013) 13:455–61. 10.1007/s10142-013-0337-0 PMC3824579 24081513

[116] Ban Y Taniyama M Ban Y. Vitamin D receptor gene polymorphisms in Hashimoto’s thyroiditis. Thyroid (2001) 11:607–8. 10.1089/105072501750302967 11442012

[117] Santos LR Durães C Mendes A Prazeres H Alvelos MI Moreira CS. A polymorphism in the promoter region of the selenoprotein S gene (SEPS1) contributes to Hashimoto’s thyroiditis susceptibility. J Clin Endocrinol Metab (2014) 99:E719–723. 10.1210/jc.2013-3539 24471570

[118] Li M Sun H Liu S Yu J Li Q Liu P. CD40 C/T-1 polymorphism plays different roles in Graves’ disease and Hashimoto’s thyroiditis: a meta-analysis. Endocr J (2012) 59:1041–50. 10.1507/endocrj.ej12-0126 22863718

[119] Zeitlin AA Heward JM Newby PR Carr-Smith JD Franklyn JA Gough SCL. Analysis of HLA class II genes in Hashimoto’s thyroiditis reveals differences compared to Graves’ disease. Genes Immun (2008) 9:358–63. 10.1038/gene.2008.26 18449200

[120] Fujii A Inoue N Watanabe M Kawakami C Hidaka Y Hayashizaki Y. TSHR Gene Polymorphisms in the Enhancer Regions Are Most Strongly Associated with the Development of Graves’ Disease, Especially Intractable Disease, and of Hashimoto’s Disease. Thyroid (2017) 27:111–9. 10.1089/thy.2016.0345 27762730

[121] Ueda H Howson JMM Esposito L Heward J Snook H Chamberlain G. Association of the T-cell regulatory gene CTLA4 with susceptibility to autoimmune disease. Nature (2003) 423:506–11. 10.1038/nature01621 12724780

[122] Furugaki K Shirasawa S Ishikawa N Ito K Ito K Kubota S. Association of the T-cell regulatory gene CTLA4 with Graves’ disease and autoimmune thyroid disease in the Japanese. J Hum Genet (2004) 49:166–8. 10.1007/s10038-003-0120-5 14986169

[123] Smyth D Cooper JD Collins JE Heward JM Franklyn JA Howson JMM. Replication of an association between the lymphoid tyrosine phosphatase locus (LYP/PTPN22) with type 1 diabetes, and evidence for its role as a general autoimmunity locus. Diabetes (2004) 53:3020–3. 10.2337/diabetes.53.11.3020 15504986

[124] Heward JM Brand OJ Barrett JC Carr-Smith JD Franklyn JA Gough SC. Association of PTPN22 haplotypes with Graves’ disease. J Clin Endocrinol Metab (2007) 92:685–90. 10.1210/jc.2006-2064 17148556

[125] Brand OJ Barrett JC Simmonds MJ Newby PR McCabe CJ Bruce CK. Association of the thyroid stimulating hormone receptor gene (TSHR) with Graves’ disease. Hum Mol Genet (2009) 18:1704–13. 10.1093/hmg/ddp087 19244275

[126] Dechairo BM Zabaneh D Collins J Brand O Dawson GJ Green AP. Association of the TSHR gene with Graves’ disease: the first disease specific locus. Eur J Hum Genet (2005) 13:1223–30. 10.1038/sj.ejhg.5201485 16106256

[127] Gao X Wang J Yu Y. The Association Between STAT4 rs7574865 Polymorphism and the Susceptibility of Autoimmune Thyroid Disease: A Meta-Analysis. Front Genet (2018) 9:708. 10.3389/fgene.2018.00708 30666271 PMC6330290

[128] Simmonds MJ. GWAS in autoimmune thyroid disease: redefining our understanding of pathogenesis. Nat Rev Endocrinol (2013) 9:277–87. 10.1038/nrendo.2013.56 23529038

[129] Wang X Cheng W Ma Y Zhu J. Vitamin D receptor gene FokI but not TaqI, ApaI, BsmI polymorphism is associated with Hashimoto’s thyroiditis: a meta-analysis. Sci Rep (2017) 7:1–11. 10.1038/srep41540 28134349 PMC5278388

[130] Wang D Chen J Zhang H Zhang F Yang L Mou Y. Role of Different CD40 Polymorphisms in Graves’ Disease and Hashimoto’s Thyroiditis. Immunol Invest (2017) 46:544–51. 10.1080/08820139.2017.1319382 28742400

[131] Ban Y Tozaki T Taniyama M Nakano Y Ban Y Ban Y. Association of the protein tyrosine phosphatase nonreceptor 22 haplotypes with autoimmune thyroid disease in the Japanese population. Thyroid (2010) 20:893–9. 10.1089/thy.2010.0104 20615141

[132] Shen X Yan X Xie B Xu D Wang K Zhu J. Genetic variants of interleukin-4 gene in autoimmune thyroid diseases: an updated meta-analysis. Autoimmunity (2015) 48:129–35. 10.3109/08916934.2014.962025 25286078

[133] Eriksson N Tung JY Kiefer AK Hinds DA Francke U Mountain JL. Novel Associations for Hypothyroidism Include Known Autoimmune Risk Loci. PLoS One (2012) 7:e34442. 10.1371/journal.pone.0034442 22493691 PMC3321023

[134] Cooper JD Simmonds MJ Walker NM Burren O Brand OJ Guo H. Seven newly identified loci for autoimmune thyroid disease. Hum Mol Genet (2012) 21:5202–8. 10.1093/hmg/dds357 PMC3490518 22922229

[135] Pickrell JK Berisa T Liu JZ Ségurel L Tung JY Hinds DA. Detection and interpretation of shared genetic influences on 42 human traits. Nat Genet (2016) 48:709–17. 10.1038/ng.3570 PMC5207801 27182965

[136] Kichaev G Bhatia G Loh P-R Gazal S Burch K Freund MK. Leveraging Polygenic Functional Enrichment to Improve GWAS Power. Am J Hum Genet (2019) 104:65–75. 10.1016/j.ajhg.2018.11.008 30595370 PMC6323418

[137] Rydzewska M Góralczyk A Gościk J Wawrusiewicz-Kurylonek N Bossowska A Krętowski A. Analysis of chosen polymorphisms rs2476601 a/G - PTPN22, rs1990760 C/T - IFIH1, rs179247 a/G - TSHR in pathogenesis of autoimmune thyroid diseases in children. Autoimmunity (2018) 51:183–90. 10.1080/08916934.2018.1486824 29973096

[138] Jabrocka-Hybel A Skalniak A Piątkowski J Turek-Jabrocka R Vyhouskaya P Ludwig-Słomczyńska A. How much of the predisposition to Hashimoto’s thyroiditis can be explained based on previously reported associations? J Endocrinol Invest (2018) 41:1409–16. 10.1007/s40618-018-0910-4 PMC6244553 29931474

[139] Medici M Porcu E Pistis G Teumer A Brown SJ Jensen RA. Identification of novel genetic Loci associated with thyroid peroxidase antibodies and clinical thyroid disease. PLoS Genet (2014) 10:e1004123. 10.1371/journal.pgen.1004123 24586183 PMC3937134

[140] Oryoji D Ueda S Yamamoto K Yoshimura Noh J Okamura K Noda M. Identification of a Hashimoto thyroiditis susceptibility locus via a genome-wide comparison with Graves’ disease. J Clin Endocrinol Metab (2015) 100:E319–324. 10.1210/jc.2014-3431 25429627

[141] Kwak SH Park YJ Go MJ Lee KE Kim S Choi HS. A genome-wide association study on thyroid function and anti-thyroid peroxidase antibodies in Koreans. Hum Mol Genet (2014) 23:4433–42. 10.1093/hmg/ddu145 PMC4103676 24722205

[142] Gudmundsson J Sulem P Gudbjartsson DF Jonasson JG Masson G He H. Discovery of common variants associated with low TSH levels and thyroid cancer risk. Nat Genet (2012) 44:319–22. 10.1038/ng.1046 PMC3655412 22267200

[143] Porcu E Medici M Pistis G Volpato CB Wilson SG Cappola AR. A meta-analysis of thyroid-related traits reveals novel loci and gender-specific differences in the regulation of thyroid function. PLoS Genet (2013) 9:e1003266. 10.1371/journal.pgen.1003266 23408906 PMC3567175

[144] Panicker V Wilson SG Walsh JP Richards JB Brown SJ Beilby JP. A locus on chromosome 1p36 is associated with thyrotropin and thyroid function as identified by genome-wide association study. Am J Hum Genet (2010) 87:430–5. 10.1016/j.ajhg.2010.08.005 PMC2933351 20826269

[145] Zhan M Chen G Pan C-M Gu Z-H Zhao S-X Liu W. Genome-wide association study identifies a novel susceptibility gene for serum TSH levels in Chinese populations. Hum Mol Genet (2014) 23:5505–17. 10.1093/hmg/ddu250 24852370

[146] Brčić L Barić A Gračan S Torlak V Brekalo M Škrabić V. Genome-wide association analysis suggests novel loci underlying thyroid antibodies in Hashimoto’s thyroiditis. Sci Rep (2019) 9:1–10. 10.1038/s41598-019-41850-6 30926877 PMC6440971

[147] Brčić L Barić A Gračan S Brdar D Torlak Lovrić V Vidan N. Association of established thyroid peroxidase autoantibody (TPOAb) genetic variants with Hashimoto’s thyroiditis. Autoimmunity (2016) 49:480–5. 10.1080/08916934.2016.1191475 27268232

[148] Matana A Popović M Boutin T Torlak V Brdar D Gunjača I. Genome-wide meta-analysis identifies novel gender specific loci associated with thyroid antibodies level in Croatians. Genomics (2019) 111:737–43. 10.1016/j.ygeno.2018.04.012 29678681

[149] Pastuszak-Lewandoska D Sewerynek E Domańska D Gładyś A Skrzypczak R Brzeziańska E. CTLA-4 gene polymorphisms and their influence on predisposition to autoimmune thyroid diseases (Graves’ disease and Hashimoto’s thyroiditis). Arch Med Sci (2012) 8:415–21. 10.5114/aoms.2012.28593 PMC3400896 22851994

[150] Ramgopal S Rathika C Padma MR Murali V Arun K Kamaludeen MN. Interaction of HLA-DRB1* alleles and CTLA4 (+49 AG) gene polymorphism in Autoimmune Thyroid Disease. Gene (2018) 642:430–8. 10.1016/j.gene.2017.11.057 29174716

[151] Ban Y Tozaki T Taniyama M Tomita M Ban Y. Association of a CTLA-4 3’ untranslated region (CT60) single nucleotide polymorphism with autoimmune thyroid disease in the Japanese population. Autoimmunity (2005) 38:151–3. 10.1080/08916930500050319 16040335

[152] Ting W-H Chien M-N Lo F-S Wang C-H Huang C-Y Lin C-L. Association of Cytotoxic T-Lymphocyte-Associated Protein 4 (CTLA4) Gene Polymorphisms with Autoimmune Thyroid Disease in Children and Adults: Case-Control Study. PLoS One (2016) 11:e0154394. 10.1371/journal.pone.0154394 27111218 PMC4844099

[153] Brčić L Gračan S Barić A Gunjača I Torlak Lovrić V Kolčić I. Association of Established Thyroid-stimulating Hormone and Free Thyroxine Genetic Variants with Hashimoto’s Thyroiditis. Immunol Invest (2017) 46:625–38. 10.1080/08820139.2017.1337785 28753406

[154] Tomer Y Dolan LM Kahaly G Divers J D’Agostino RB Imperatore G. Genome wide identification of new genes and pathways in patients with both autoimmune thyroiditis and type 1 diabetes. J Autoimmun (2015) 60:32–9. 10.1016/j.jaut.2015.03.006 PMC4457545 25936594

[155] Arnaud-Lopez L Usala G Ceresini G Mitchell BD Pilia MG Piras MG. Phosphodiesterase 8B Gene Variants Are Associated with Serum TSH Levels and Thyroid Function. Am J Hum Genet (2008) 82:1270–80. 10.1016/j.ajhg.2008.04.019 PMC2427267 18514160

[156] Barić A Brčić L Gračan S Torlak Lovrić V Gunjača I Šimunac M. Association of established hypothyroidism-associated genetic variants with Hashimoto’s thyroiditis. J Endocrinol Invest (2017) 40:1061–7. 10.1007/s40618-017-0660-8 28382505

[157] Brčić L Barić A Gračan S Brekalo M Kaličanin D Gunjača I. Genome-wide association analysis suggests novel loci for Hashimoto’s thyroiditis. J Endocrinol Invest (2019) 42:567–76. 10.1007/s40618-018-0955-4 30284222

[158] Denny JC Crawford DC Ritchie MD Bielinski SJ Basford MA Bradford Y. Variants near FOXE1 are associated with hypothyroidism and other thyroid conditions: using electronic medical records for genome- and phenome-wide studies. Am J Hum Genet (2011) 89:529–42. 10.1016/j.ajhg.2011.09.008 PMC3188836 21981779

[159] Wang B Jia X Yao Q Li Q He W Li L. CEP128 is a crucial risk locus for autoimmune thyroid diseases. Mol Cell Endocrinol (2019) 480:97–106. 10.1016/j.mce.2018.10.017 30393005

[160] Matana A Boutin T Torlak V Brdar D Gunjača I Kolčić I. Genome-Wide Analysis Identifies Two Susceptibility Loci for Positive Thyroid Peroxidase and Thyroglobulin Antibodies. J Clin Endocrinol Metab (2020) 105:dgz239. 10.1210/clinem/dgz239 31794020

[161] Hwangbo Y Park YJ. Genome-Wide Association Studies of Autoimmune Thyroid Diseases, Thyroid Function, and Thyroid Cancer. Endocrinol Metab (2018) 33:175–84. 10.3803/EnM.2018.33.2.175 PMC6021314 29947174

[162] Gudmundsson J Sulem P Gudbjartsson DF Jonasson JG Sigurdsson A Bergthorsson JT. Common variants on 9q22.33 and 14q13.3 predispose to thyroid cancer in European populations. Nat Genet (2009) 41:460–4. 10.1038/ng.339 PMC3664837 19198613

[163] Castanet M Polak M. Spectrum of Human Foxe1/TTF2 Mutations. Horm Res Paediatr (2010) 73:423–9. 10.1159/000281438 20453517

[164] Fagman H Nilsson M. Morphogenetics of early thyroid development. J Mol Endocrinol (2011) 46:R33–42. 10.1677/jme-10-0084 21322126

[165] Ortiz L Aza-Blanc P Zannini M Cato AC Santisteban P. The interaction between the forkhead thyroid transcription factor TTF-2 and the constitutive factor CTF/NF-1 is required for efficient hormonal regulation of the thyroperoxidase gene transcription. J Biol Chem (1999) 274:15213–21. 10.1074/jbc.274.21.15213 10329730

[166] Bender AT Beavo JA. Cyclic nucleotide phosphodiesterases: molecular regulation to clinical use. Pharmacol Rev (2006) 58:488–520. 10.1124/pr.58.3.5 16968949

[167] Dumont C Corsoni-Tadrzak A Ruf S de Boer J Williams A Turner M. Rac GTPases play critical roles in early T-cell development. Blood (2009) 113:3990–8. 10.1182/blood-2008-09-181180 PMC2673125 19088377

[168] Malhotra S Kovats S Zhang W Coggeshall KM. B Cell Antigen Receptor Endocytosis and Antigen Presentation to T Cells Require Vav and Dynamin. J Biol Chem (2009) 284:24088–97. 10.1074/jbc.M109.014209 PMC2782002 19586920

[169] Sasazuki T Inoko H Morishima S Morishima Y. Gene Map of the HLA Region, Graves’ Disease and Hashimoto Thyroiditis, and Hematopoietic Stem Cell Transplantation. Adv Immunol (2016) 129:175–249. 10.1016/bs.ai.2015.08.003 26791860

[170] Ueda S Oryoji D Yamamoto K Noh JY Okamura K Noda M. Identification of independent susceptible and protective HLA alleles in Japanese autoimmune thyroid disease and their epistasis. J Clin Endocrinol Metab (2014) 99:E379–383. 10.1210/jc.2013-2841 24285682

[171] Bernecker C Ostapczuk M Vordenbäumen S Ehlers M Thiel A Schinner S. HLA-A2 phenotype may be protective against Graves’ disease but not against Hashimoto’s thyroiditis in Caucasians. Horm Metab Res (2013) 45:74–7. 10.1055/s-0032-1323704 22972181

[172] Tomer Y Davies TF. Searching for the autoimmune thyroid disease susceptibility genes: from gene mapping to gene function. Endocr Rev (2003) 24:694–717. 10.1210/er.2002-0030 14570752

[173] Tendon N Zhang L Weetman AP. HLA Associations with Hashimoto’s thyroiditis. Clin Endocrinol (Oxf) (1991) 34:383–6. 10.1111/j.1365-2265.1991.tb00309.x 1676351

[174] Shi Y Zou M Robb D Farid NR. Typing for major histocompatibility complex class II antigens in thyroid tissue blocks: association of Hashimoto’s thyroiditis with HLA-DQA0301 and DQB0201 alleles. J Clin Endocrinol Metab (1992) 75:943–6. 10.1210/jcem.75.3.1517390 1517390

[175] Hunt PJ Marshall SE Weetman AP Bunce M Bell JI Wass JA. Histocompatibility leucocyte antigens and closely linked immunomodulatory genes in autoimmune thyroid disease. Clin Endocrinol (Oxf) (2001) 55:491–9. 10.1046/j.1365-2265.2001.01356.x 11678832

[176] Petrone A Giorgi G Mesturino CA Capizzi M Cascino I Nistico L. Association of DRB1*04-DQB1*0301 haplotype and lack of association of two polymorphic sites at CTLA-4 gene with Hashimoto’s thyroiditis in an Italian population. Thyroid (2001) 11:171–5. 10.1089/105072501300042901 11288988

[177] Kokaraki G Daniilidis M Yiangou M Arsenakis M Karyotis N Tsilipakou M. Major histocompatibility complex class II (DRB1*, DQA1*, and DQB1*) and DRB1*04 subtypes’ associations of Hashimoto’s thyroiditis in a Greek population. Tissue Antigens (2009) 73:199–205. 10.1111/j.1399-0039.2008.01182.x 19254248

[178] Cho WK Jung MH Choi E-J Choi H-B Kim T-G Suh B-K. Association of HLA Alleles with Autoimmune Thyroid Disease in Korean Children. Horm Res Paediatr (2011) 76:328–34. 10.1159/000331134 21952423

[179] Patiroğlu T Akar HH Akay A Ok E. The distribution of HLA-DRB1 alleles in patients with hashimoto’s thyroiditis -. Şişli Etfal Tıp Bül (2015) 49:255–9. 10.5350/SEMB.20151005010345

[180] Ban Y Davies TF Greenberg DA Concepcion ES Osman R Oashi T. Arginine at position 74 of the HLA-DR beta1 chain is associated with Graves’ disease. Genes Immun (2004) 5:203–8. 10.1038/sj.gene.6364059 15029234

[181] Menconi F Monti MC Greenberg DA Oashi T Osman R Davies TF. Molecular amino acid signatures in the MHC class II peptide-binding pocket predispose to autoimmune thyroiditis in humans and in mice. Proc Natl Acad Sci U S A (2008) 105:14034–9. 10.1073/pnas.0806584105 PMC2544574 18779568

[182] Hodge SE Ban Y Strug LJ Greenberg DA Davies TF Concepcion ES. Possible Interaction Between HLA-DRβ1 and Thyroglobulin Variants in Graves’ Disease. Thyroid (2006) 16:351–5. 10.1089/thy.2006.16.351 16646680

[183] Ni J Qiu L-J Zhang M Wen P-F Ye X-R Liang Y. CTLA-4 CT60 (rs3087243) polymorphism and autoimmune thyroid diseases susceptibility: a comprehensive meta-analysis. Endocr Res (2014) 39:180–8. 10.3109/07435800.2013.879167 24697361

[184] Hou H-F Jin X Sun T Li C Jiang B-F Li Q-W. Cytotoxic T Lymphocyte-Associated Antigen 4 Gene Polymorphisms and Autoimmune Thyroid Diseases: An Updated Systematic Review and Cumulative Meta-Analysis. Int J Endocrinol (2015) 2015:747816. 10.1155/2015/747816 25878663 PMC4387902

[185] Ji R Feng Y Zhan WW. Updated analysis of studies on the cytotoxic T-lymphocyte-associated antigen-4 gene A49G polymorphism and Hashimoto’s thyroiditis risk. Genet Mol Res (2013) 12:1421–30. 10.4238/2013.April.26.4 23661465

[186] Hu Y Xu K Jiang L Zhang L Shi H Cui D. Associations Between Three CTLA-4 Polymorphisms and Hashimoto’s Thyroiditis Risk: An Updated Meta-Analysis with Trial Sequential Analysis. Genet Test Mol Biomarkers (2018) 22:224–36. 10.1089/gtmb.2017.0243 29461867

[187] Qiu H Tang W Yin P Cheng F Wang L. Cytotoxic T-lymphocyte associated antigen 4 polymorphism and Hashimoto’s thyroiditis susceptibility: a meta-analysis. Endocrine (2014) 45:198–205. 10.1007/s12020-013-9985-z 23677500

[188] Vieland VJ Huang Y Bartlett C Davies TF Tomer Y. A multilocus model of the genetic architecture of autoimmune thyroid disorder, with clinical implications. Am J Hum Genet (2008) 82:1349–56. 10.1016/j.ajhg.2008.04.017 PMC2427261 18485327

[189] Einarsdottir E Söderström I Löfgren-Burström A Haraldsson S Nilsson-Ardnor S Penha-Goncalves C. The CTLA4 region as a general autoimmunity factor: an extended pedigree provides evidence for synergy with the HLA locus in the etiology of type 1 diabetes mellitus, Hashimoto’s thyroiditis and Graves’ disease. Eur J Hum Genet EJHG (2003) 11:81–4. 10.1038/sj.ejhg.5200903 12529710

[190] Teft WA Kirchhof MG Madrenas J. A molecular perspective of CTLA-4 function. Annu Rev Immunol (2006) 24:65–97. 10.1146/annurev.immunol.24.021605.090535 16551244

[191] Xiaoheng C Yizhou M Bei H Huilong L Xin W Rui H. General and Specific Genetic Polymorphism of Cytokines-Related Gene in AITD. Mediators Inflamm (2017) 2017:e3916395. 10.1155/2017/3916395 PMC5241475 28133421

[192] Ban Y Greenberg DA Davies TF Jacobson E Concepcion E Tomer Y. ‘Linkage Analysis of Thyroid Antibody Production: Evidence for Shared Susceptibility to Clinical Autoimmune Thyroid Disease. J Clin Endocrinol Metab (2008) 93:3589–96. 10.1210/jc.2008-0364 PMC2567858 18559906

[193] Cloutier JF Veillette A. Cooperative inhibition of T-cell antigen receptor signaling by a complex between a kinase and a phosphatase. J Exp Med (1999) 189:111–21. 10.1084/jem.189.1.111 PMC1887684 9874568

[194] Hill RJ Zozulya S Lu Y-L Ward K Gishizky M Jallal B. The lymphoid protein tyrosine phosphatase Lyp interacts with the adaptor molecule Grb2 and functions as a negative regulator of T-cell activation. Exp Hematol (2002) 30:237–44. 10.1016/S0301-472X(01)00794-9 11882361

[195] Burn GL Svensson L Sanchez-Blanco C Saini M Cope AP. Why is PTPN22 a good candidate susceptibility gene for autoimmune disease? FEBS Lett (2011) 585:3689–98. 10.1016/j.febslet.2011.04.032 21515266

[196] Velaga MR Wilson V Jennings CE Owen CJ Herington S Donaldson PT. The codon 620 tryptophan allele of the lymphoid tyrosine phosphatase (LYP) gene is a major determinant of Graves’ disease. J Clin Endocrinol Metab (2004) 89:5862–5. 10.1210/jc.2004-1108 15531553

[197] Criswell LA Pfeiffer KA Lum RF Gonzales B Novitzke J Kern M. Analysis of families in the multiple autoimmune disease genetics consortium (MADGC) collection: the PTPN22 620W allele associates with multiple autoimmune phenotypes. Am J Hum Genet (2005) 76:561–71. 10.1086/429096 PMC1199294 15719322

[198] Ban Y Tozaki T Taniyama M Tomita M Ban Y. The codon 620 single nucleotide polymorphism of the protein tyrosine phosphatase-22 gene does not contribute to autoimmune thyroid disease susceptibility in the Japanese. Thyroid (2005) 15:1115–8. 10.1089/thy.2005.15.1115 16279843

[199] Chabchoub G Teixiera EP Maalej A Ben Hamad M Bahloul Z Cornelis F. The R620W polymorphism of the protein tyrosine phosphatase 22 gene in autoimmune thyroid diseases and rheumatoid arthritis in the Tunisian population. Ann Hum Biol (2009) 36:342–9. 10.1080/03014460902817968 19343596

[200] Luo L Cai B Liu F Hu X Wang L. Association of Protein Tyrosine Phosphatase Nonreceptor 22 (PTPN22) C1858T gene polymorphism with susceptibility to autoimmune thyroid diseases: a meta-analysis. Endocr J (2012) 59:439–45. 10.1507/endocrj.ej11-0381 22374238

[201] Alkhateeb A Marzouka NA-D Tashtoush R. Variants in PTPN22 and SMOC2 genes and the risk of thyroid disease in the Jordanian Arab population. Endocrine (2013) 44:702–9. 10.1007/s12020-013-9908-z 23463390

[202] Mori M Yamada R Kobayashi K Kawaida R Yamamoto K. Ethnic differences in allele frequency of autoimmune-disease-associated SNPs. J Hum Genet (2005) 50:264–6. 10.1007/s10038-005-0246-8 15883854

[203] Reddy MVPL Johansson M Sturfelt G Jönsen A Gunnarsson I Svenungsson E. The R620W C/T polymorphism of the gene PTPN22 is associated with SLE independently of the association of PDCD1. Genes Immun (2005) 6:658–62. 10.1038/sj.gene.6364252 16052172

[204] Gong L Liu B Wang J Pan H Qi A Zhang S. Novel missense mutation in PTPN22 in a Chinese pedigree with Hashimoto’s thyroiditis. BMC Endocr Disord (2018) 18:1–6. 10.1186/s12902-018-0305-8 30384852 PMC6211547

[205] Bottini N Musumeci L Alonso A Rahmouni S Nika K Rostamkhani M. A functional variant of lymphoid tyrosine phosphatase is associated with type I diabetes. Nat Genet (2004) 36:337–8. 10.1038/ng1323 15004560

[206] Gianchecchi E Palombi M Fierabracci A. The putative role of the C1858T polymorphism of protein tyrosine phosphatase PTPN22 gene in autoimmunity. Autoimmun Rev (2013) 12:717–25. 10.1016/j.autrev.2012.12.003 23261816

[207] Ferreira RC Castro Dopico X Oliveira JJ Rainbow DB Yang JH Trzupek D. Chronic Immune Activation in Systemic Lupus Erythematosus and the Autoimmune PTPN22 Trp620 Risk Allele Drive the Expansion of FOXP3+ Regulatory T Cells and PD-1 Expression. Front Immunol (2019) 10:2606. 10.3389/fimmu.2019.02606 31781109 PMC6857542

[208] Rawlings DJ Dai X Buckner JH. The role of PTPN22 risk variant in the development of autoimmunity: Finding common ground between mouse and man. J Immunol Baltim Md 1950 (2015) 194:2977–84. 10.4049/jimmunol.1403034 PMC4369788 25795788

[209] Weetman AP. The Immunopathogenesis of Chronic Autoimmune Thyroiditis One Century after Hashimoto. Eur Thyroid J (2013) 1:243–50. 10.1159/000343834 PMC3821488 24783026

[210] Janssen OE Mehlmauer N Hahn S Offner AH Gartner R. High prevalence of autoimmune thyroiditis in patients with polycystic ovary syndrome. Eur J Endocrinol (2004) 150:363–9. 10.1530/eje.0.1500363 15012623

[211] Anaforoglu I Topbas M Algun E. Relative associations of polycystic ovarian syndrome vs metabolic syndrome with thyroid function, volume, nodularity and autoimmunity. J Endocrinol Invest (2011) 34:e259–264. 10.3275/7681 21521934

[212] Kachuei M Jafari F Kachuei A Keshteli AH. Prevalence of autoimmune thyroiditis in patients with polycystic ovary syndrome. Arch Gynecol Obstet (2012) 285:853–6. 10.1007/s00404-011-2040-5 21866332

[213] Sinha U Sinharay K Saha S Longkumer TA Baul SN Pal SK. Thyroid disorders in polycystic ovarian syndrome subjects: A tertiary hospital based cross-sectional study from Eastern India. Indian J Endocrinol Metab (2013) 17:304. 10.4103/2230-8210.109714 23776908 PMC3683210

[214] Garelli S Masiero S Plebani M Chen S Furmaniak J Armanini D. High prevalence of chronic thyroiditis in patients with polycystic ovary syndrome. Eur J Obstet Gynecol Reprod Biol (2013) 169:248–51. 10.1016/j.ejogrb.2013.03.003 23548659

[215] Al-Saab R Haddad S. Detection of Thyroid Autoimmunity Markers in Euthyroid Women With Polycystic Ovary Syndrome: A Case-Control Study From Syria. Int J Endocrinol Metab (2014) 12:e17954. 10.5812/ijem.17954 25237328 PMC4166006

[216] Duran C Basaran M Kutlu O Kucukaydin Z Bakdik S Burnik FS. Frequency of nodular goiter and autoimmune thyroid disease in patients with polycystic ovary syndrome. Endocrine (2015) 49:464–9. 10.1007/s12020-014-0504-7 25522724

[217] Petrikova J Lazurova I Dravecka I Vrbikova J Kozakova D Figurova J. The prevalence of non organ specific and thyroid autoimmunity in patients with polycystic ovary syndrome. BioMed Pap Med Fac Univ Palacky Olomouc Czech Repub (2015) 159:302–6. 10.5507/bp.2014.062 25485530

[218] Calvar CE Bengolea SV Deutsch SI Hermes R Ramos G Loyato M. [High frequency of thyroid abnormalities in polycystic ovary syndrome]. Medicina (Mex) (2015) 75:213–7. 26339875

[219] Arduc A Aycicek Dogan B Bilmez S Imga Nasiroglu N Tuna MM Isik S. High prevalence of Hashimoto’s thyroiditis in patients with polycystic ovary syndrome: does the imbalance between estradiol and progesterone play a role? Endocr Res (2015) 40:204–10. 10.3109/07435800.2015.1015730 25822940

[220] Yu Q Wang J-B. Subclinical Hypothyroidism in PCOS: Impact on Presentation, Insulin Resistance, and Cardiovascular Risk. BioMed Res Int (2016) 2016:2067087. 10.1155/2016/2067087 27478827 PMC4960326

[221] Arora S Sinha K Kolte S Mandal A. Endocrinal and autoimmune linkage: Evidences from a controlled study of subjects with polycystic ovarian syndrome. J Hum Reprod Sci (2016) 9:18–22. 10.4103/0974-1208.178636 27110073 PMC4817282

[222] Menon M Ramachandran V. Antithyroid Peroxidase Antibodies in Women with Polycystic Ovary Syndrome. J Obstet Gynaecol India (2017) 67:61–5. 10.1007/s13224-016-0914-y PMC5306098 28242970

[223] Karaköse M Hepsen S Çakal E Saykı Arslan M Tutal E Akın Ş. Frequency of nodular goiter and autoimmune thyroid disease and association of these disorders with insulin resistance in polycystic ovary syndrome. J Turk Ger Gynecol Assoc (2017) 18:85–9. 10.4274/jtgga.2016.0217 PMC5458441 28400351

[224] Yin D Ruan X Tian X Du J Zhao Y Cui Y. The relationship between thyroid function and metabolic changes in Chinese women with polycystic ovary syndrome. Gynecol Endocrinol (2017) 33:332–5. 10.1080/09513590.2016.1273895 28051891

[225] Hepşen S Karaköse M Çakal E Öztekin S Ünsal İ Akhanlı P. The assessment of thyroid autoantibody levels in euthyroid patients with polycystic ovary syndrome. J Turk Ger Gynecol Assoc (2018) 19:215–9. 10.4274/jtgga.2018.0001 PMC6250086 29699958

[226] Ulrich J Goerges J Keck C Müller-Wieland D Diederich S Janssen OE. Impact of Autoimmune Thyroiditis on Reproductive and Metabolic Parameters in Patients with Polycystic Ovary Syndrome. Exp Clin Endocrinol Diabetes (2018) 126:198–204. 10.1055/s-0043-110480 29506313

[227] Enzevaei A Salehpour S Tohidi M Saharkhiz N. Subclinical hypothyroidism and insulin resistance in polycystic ovary syndrome: is there a relationship? Iran J Reprod Med (2014) 12:481–6. PMC4126252 25114670

[228] Bedaiwy MA Abdel-Rahman MY Tan J AbdelHafez FF Abdelkareem AO Henry D. Clinical, Hormonal, and Metabolic Parameters in Women with Subclinical Hypothyroidism and Polycystic Ovary Syndrome: A Cross-Sectional Study. J Womens Health (2018) 27:659–64. 10.1089/jwh.2017.6584 29620956

[229] Ding X Yang L Wang J Tang R Chen Q Pan J. Subclinical Hypothyroidism in Polycystic Ovary Syndrome: A Systematic Review and Meta-Analysis. Front Endocrinol (2018) 9:700. 10.3389/fendo.2018.00700 PMC6277795 30542323

[230] Romitti M Fabris VC Ziegelmann PK Maia AL Spritzer PM. Association between PCOS and autoimmune thyroid disease: a systematic review and meta-analysis. Endocr Connect (2018) 7:1158–67. 10.1530/EC-18-0309 PMC6215798 30352422

[231] Hefler-Frischmuth K Walch K Huebl W Baumuehlner K Tempfer C Hefler L. Serologic markers of autoimmunity in women with polycystic ovary syndrome. Fertil Steril (2010) 93:2291–4. 10.1016/j.fertnstert.2009.01.056 19296936

[232] Makled AK Fathi HM Gomaa MF Bakr RM. Serologic markers of autoimmunity in women with polycystic ovary syndrome. Middle East Fertil Soc J (2015) 20:86–90. 10.1016/j.mefs.2014.05.006

[233] Kowalczyk K Franik G Kowalczyk D Pluta D Blukacz Ł Madej P. Thyroid disorders in polycystic ovary syndrome. Eur Rev Med Pharmacol Sci (2017) 21:346–60. 28165551

[234] de Medeiros SF de Medeiros MAS Ormond CM Barbosa JS Yamamoto MMW. Subclinical Hypothyroidism Impact on the Characteristics of Patients with Polycystic Ovary Syndrome. A Meta-Analysis of Observational Studies. Gynecol Obstet Invest (2018) 83:105–15. 10.1159/000485619 30025406

[235] Zhang B Wang J Shen S Liu J Sun J Gu T. Subclinical hypothyroidism is not a risk factor for polycystic ovary syndrome in obese women of reproductive age. Gynecol Endocrinol (2018) 34:875–9. 10.1080/09513590.2018.1462319 29658805

[236] Muscogiuri G Sorice GP Mezza T Prioletta A Lassandro AP Pirronti T. High-normal TSH values in obesity: is it insulin resistance or adipose tissue’s guilt? Obesity Silver Spring Md (2013) 21:101–6. 10.1002/oby.20240 23505173

[237] Tagliaferri V Romualdi D Guido M Mancini A De Cicco S Di Florio C. The link between metabolic features and TSH levels in polycystic ovary syndrome is modulated by the body weight: an euglycaemic-hyperinsulinaemic clamp study. Eur J Endocrinol (2016) 175:433–41. 10.1530/EJE-16-0358 27511825

[238] Chapman JC Min SH Freeh SM Michael SD. The estrogen-injected female mouse: new insight into the etiology of PCOS. Reprod Biol Endocrinol (2009) 7:47. 10.1186/1477-7827-7-47 19450261 PMC2695461

[239] Chen W Jin W Hardegen N Lei K-J Li L Marinos N. Conversion of peripheral CD4+CD25- naive T cells to CD4+CD25+ regulatory T cells by TGF-beta induction of transcription factor Foxp3. J Exp Med (2003) 198:1875–86. 10.1084/jem.20030152 PMC2194145 14676299

[240] Akinci B Comlekci A Yener S Bayraktar F Demir T Ozcan MA. Hashimoto’s thyroiditis, but not treatment of hypothyroidism, is associated with altered TGF-beta1 levels. Arch Med Res (2008) 39:397–401. 10.1016/j.arcmed.2007.12.001 18375250

[241] Govinden R Bhoola KD. Genealogy, expression, and cellular function of transforming growth factor-β. Pharmacol Ther (2003) 98:257–65. 10.1016/S0163-7258(03)00035-4 12725873

[242] Raja-Khan N Urbanek M Rodgers RJ Legro RS. The role of TGF-β in polycystic ovary syndrome. Reprod Sci Thousand Oaks Calif (2014) 21:20–31. 10.1177/1933719113485294 PMC5933191 23585338

[243] Quintero OL Amador-Patarroyo MJ Montoya-Ortiz G Rojas-Villarraga A Anaya J-M. Autoimmune disease and gender: plausible mechanisms for the female predominance of autoimmunity. J Autoimmun (2012) 38:J109–119. 10.1016/j.jaut.2011.10.003 22079680

[244] Pennell LM Galligan CL Fish EN. Sex affects immunity. J Autoimmun (2012) 38:J282–291. 10.1016/j.jaut.2011.11.013 22225601

[245] Moulton VR. Sex Hormones in Acquired Immunity and Autoimmune Disease. Front Immunol (2018) 9:1–21. 10.3389/fimmu.2018.02279 30337927 PMC6180207

[246] Angstwurm MW Gärtner R Ziegler-Heitbrock HW. Cyclic plasma IL-6 levels during normal menstrual cycle. Cytokine (1997) 9:370–4. 10.1006/cyto.1996.0178 9195137

[247] Zhou X Bailey-Bucktrout S Jeker LT Bluestone JA. Plasticity of CD4(+) FoxP3(+) T cells. Curr Opin Immunol (2009) 21:281–5. 10.1016/j.coi.2009.05.007 PMC2733784 19500966

[248] Hughes GC. Progesterone and autoimmune disease. Autoimmun Rev (2012) 11:A502–514. 10.1016/j.autrev.2011.12.003 PMC3431799 22193289

[249] Gubbels Bupp MR Jorgensen TN. Androgen-Induced Immunosuppression. Front Immunol (2018) 9:794. 10.3389/fimmu.2018.00794 29755457 PMC5932344

[250] Petríková J Lazúrová I Yehuda S. Polycystic ovary syndrome and autoimmunity. Eur J Intern Med (2010) 21:369–71. 10.1016/j.ejim.2010.06.008 20816585

[251] Escobar-Morreale HF Luque-Ramírez M González F. Circulating Inflammatory Markers in Polycystic Ovary Syndrome: A Systematic Review and Meta-Analysis. Fertil Steril (2011) 95:1048–58.e1-2. 10.1016/j.fertnstert.2010.11.036 21168133 PMC3079565

[252] Cai J Zhang Y Wang Y Li S Wang L Zheng J. High Thyroid Stimulating Hormone Level Is Associated With Hyperandrogenism in Euthyroid Polycystic Ovary Syndrome (PCOS) Women, Independent of Age, BMI, and Thyroid Autoimmunity: A Cross-Sectional Analysis. Front Endocrinol (2019) 10:222. 10.3389/fendo.2019.00222 PMC6467931 31024459

[253] Muscogiuri G Palomba S Caggiano M Tafuri D Colao A Orio F. Low 25 (OH) vitamin D levels are associated with autoimmune thyroid disease in polycystic ovary syndrome. Endocrine (2016) 53:538–42. 10.1007/s12020-015-0745-0 26433740

[254] Tamer G Arik S Tamer I Coksert D. Relative vitamin D insufficiency in Hashimoto’s thyroiditis. Thyroid (2011) 21:891–6. 10.1089/thy.2009.0200 21751884

[255] Skaaby T Husemoen LLN Thuesen BH Linneberg A. Prospective population-based study of the association between vitamin D status and incidence of autoimmune disease. Endocrine (2015) 50:231–8. 10.1007/s12020-015-0547-4 25666936

[256] Kim D. Low vitamin D status is associated with hypothyroid Hashimoto’s thyroiditis. Hormomes Athens Greece (2016) 15:385–93. 10.14310/horm.2002.1681 27394703

[257] Nandi A Sinha N Ong E Sonmez H Poretsky L. Is there a role for vitamin D in human reproduction? Horm Mol Biol Clin Investig (2016) 25:15–28. 10.1515/hmbci-2015-0051 26943610

[258] Kim C-Y Lee YJ Choi J-H Lee SY Lee HY Jeong DH. The Association between Low Vitamin D Status and Autoimmune Thyroid Disease in Korean Premenopausal Women: The 6th Korea National Health and Nutrition Examination Survey, 2013-2014. Korean J Fam Med (2019) 40:323–8. 10.4082/kjfm.18.0075 PMC6768840 31476853

[259] Lopez ER Zwermann O Segni M Meyer G Reincke M Seissler J. A promoter polymorphism of the CYP27B1 gene is associated with Addison’s disease, Hashimoto’s thyroiditis, Graves’ disease and type 1 diabetes mellitus in Germans. Eur J Endocrinol (2004) 151:193–7. 10.1530/eje.0.1510193 15296474

[260] De Pergola G Triggiani V Bartolomeo N Giagulli VA Anelli M Masiello M. Low 25 Hydroxyvitamin D Levels are Independently Associated with Autoimmune Thyroiditis in a Cohort of Apparently Healthy Overweight and Obese Subjects. Endocr Metab Immune Disord Drug Targets (2018) 18:646–52. 10.2174/1871530318666180406163426 29623823

[261] Tsuchiya Y Nakajima M Kyo S Kanaya T Inoue M Yokoi T. Human CYP1B1 is regulated by estradiol via estrogen receptor. Cancer Res (2004) 64:3119–25. 10.1158/0008-5472.can-04-0166 15126349

[262] Jansen E Laven JSE Dommerholt HBR Polman J van Rijt C van den Hurk C. Abnormal gene expression profiles in human ovaries from polycystic ovary syndrome patients. Mol Endocrinol Baltim Md (2004) 18:3050–63. 10.1210/me.2004-0074 15308691

[263] Zou S Sang Q Wang H Feng R Li Q Zhao X. Common genetic variation in CYP1B1 is associated with concentrations of T_4_, FT_4_ and FT_4_ in the sera of polycystic ovary syndrome patients. Mol Biol Rep (2013) 40:3315–20. 10.1007/s11033-012-2406-1 23283740

[264] Marshall JC Eagleson CA. Neuroendocrine aspects of polycystic ovary syndrome. Endocrinol Metab Clin North Am (1999) 28:295–324. 10.1016/s0889-8529(05)70071-2 10352920

[265] Okada R Kobayashi T Yamamoto K Nakakura T Tanaka S Vaudry H. Neuroendocrine regulation of thyroid-stimulating hormone secretion in amphibians. Ann N Y Acad Sci (2009) 1163:262–70. 10.1111/j.1749-6632.2008.03662.x 19456347

[266] Li Q Yang G Wang Y Zhang X Sang Q Wang H. Common genetic variation in the 3’-untranslated region of gonadotropin-releasing hormone receptor regulates gene expression in cella and is associated with thyroid function, insulin secretion as well as insulin sensitivity in polycystic ovary syndrome patients. Hum Genet (2011) 129:553–61. 10.1007/s00439-011-0954-4 21274726

[267] Michalaki MA Vagenakis AG Leonardou AS Argentou MN Habeos IG Makri MG. Thyroid function in humans with morbid obesity. Thyroid (2006) 16:73–8. 10.1089/thy.2006.16.73 16487017

[268] Goodarzi MO Louwers YV Taylor KD Jones MR Cui J Kwon S. Replication of association of a novel insulin receptor gene polymorphism with polycystic ovary syndrome. Fertil Steril (2011) 95:1736–41.e1-11. 10.1016/j.fertnstert.2011.01.015 21300347 PMC3062664

[269] Song DK Hong YS Sung Y-A Lee H. Insulin resistance according to β-cell function in women with polycystic ovary syndrome and normal glucose tolerance. PLoS One (2017) 12:e0178120. 10.1371/journal.pone.0178120 28542421 PMC5444780

[270] Behre HM Greb RR Mempel A Sonntag B Kiesel L Kaltwasser P. Significance of a common single nucleotide polymorphism in exon 10 of the follicle-stimulating hormone (FSH) receptor gene for the ovarian response to FSH: a pharmacogenetic approach to controlled ovarian hyperstimulation. Pharmacogenet Genomics (2005) 15:451–6. 10.1097/01.fpc.0000167330.92786.5e 15970792

[271] Mutharasan P Galdones E Peñalver Bernabé B Garcia OA Jafari N Shea LD. Evidence for chromosome 2p16.3 polycystic ovary syndrome susceptibility locus in affected women of European ancestry. J Clin Endocrinol Metab (2013) 98:E185–190. 10.1210/jc.2012-2471 PMC3537106 23118426

[272] Weng X Ma X Wang Q Xu K Hu X Liu W. Effect of hypothyroidism on CYP51 and FSHR expression in rat ovary. Theriogenology (2019) 138:145–51. 10.1016/j.theriogenology.2019.07.012 31352176

[273] Yang J Zhong T Xiao G Chen Y Liu J Xia C. Polymorphisms and haplotypes of the TGF-β1 gene are associated with risk of polycystic ovary syndrome in Chinese Han women. Eur J Obstet Gynecol Reprod Biol (2015) 186:1–7. 10.1016/j.ejogrb.2014.11.004 25594618

[274] Urbanek M Sam S Legro RS Dunaif A. Identification of a polycystic ovary syndrome susceptibility variant in fibrillin-3 and association with a metabolic phenotype. J Clin Endocrinol Metab (2007) 92:4191–8. 10.1210/jc.2007-0761 17785364

[275] Prodoehl MJ Hatzirodos N Irving-Rodgers HF Zhao ZZ Painter JN Hickey TE. Genetic and gene expression analyses of the polycystic ovary syndrome candidate gene fibrillin-3 and other fibrillin family members in human ovaries. Mol Hum Reprod (2009) 15:829–41. 10.1093/molehr/gap072 PMC2776474 19692420

[276] Xie G Xu P Che Y Xia Y Cao Y Wang W. Microsatellite polymorphism in the fibrillin 3 gene and susceptibility to PCOS: a case-control study and meta-analysis. Reprod BioMed Online (2013) 26:168–74. 10.1016/j.rbmo.2012.10.014 23265956

[277] Raja-Khan N Kunselman AR Demers LM Ewens KG Spielman RS Legro RS. A variant in the fibrillin-3 gene is associated with TGF-β and inhibin B levels in women with polycystic ovary syndrome. Fertil Steril (2010) 94:2916–9. 10.1016/j.fertnstert.2010.05.047 PMC2989416 20630504

[278] Hatzirodos N Bayne RA Irving-Rodgers HF Hummitzsch K Sabatier L Lee S. Linkage of regulators of TGF-β activity in the fetal ovary to polycystic ovary syndrome. FASEB J (2011) 25:2256–65. 10.1096/fj.11-181099 PMC3219214 21411746

[279] Joo YY Actkins K Pacheco JA Basile AO Carroll R Crosslin DR. A Polygenic and Phenotypic Risk Prediction for Polycystic Ovary Syndrome Evaluated by Phenome-Wide Association Studies. J Clin Endocrinol Metab (2020) 105:1918–36. 10.1210/clinem/dgz326 PMC7453038 31917831

[280] Winer DA Winer S Shen L Chng MHY Engleman EG. B lymphocytes as emerging mediators of insulin resistance. Int J Obes Suppl (2012) 2:S4–7. 10.1038/ijosup.2012.2 PMC4109086 25089193

[281] Shevach EM. Regulatory T cells in autoimmmunity*. Annu Rev Immunol (2000) 18:423–49. 10.1146/annurev.immunol.18.1.423 10837065

[282] Yang M Su L Tao Q Zhang C Wu Y Liu J. Depletion of Regulatory T Cells in Visceral Adipose Tissues Contributes to Insulin Resistance in Hashimoto’s Thyroiditis. Front Physiol (2018) 9:136. 10.3389/fphys.2018.00136 29541033 PMC5835527

[283] Yin L Zeng C Yao J Shen J. Emerging Roles for Noncoding RNAs in Autoimmune Thyroid Disease. Endocrinology (2020) 161:bqaa053. 10.1210/endocr/bqaa053 32270194

[284] Lin L Du T Huang J Huang L-L Yang D-Z. Identification of differentially expressed microRNAs in the ovary of polycystic ovary syndrome with hyperandrogenism and insulin resistance. Chin Med J (Engl) (2015) 128:169–74. 10.4103/0366-6999.149189 PMC4837833 25591557

[285] Wang B Shao X Song R Xu D Zhang J-A. The Emerging Role of Epigenetics in Autoimmune Thyroid Diseases. Front Immunol (2017) 8:396. 10.3389/fimmu.2017.00396 28439272 PMC5383710

[286] Dorris ER Smyth P O’Leary JJ Sheils O. MIR141 Expression Differentiates Hashimoto Thyroiditis from PTC and Benign Thyrocytes in Irish Archival Thyroid Tissues. Front Endocrinol (2012) 3:102. 10.3389/fendo.2012.00102 PMC3432448 22969748

[287] Peng H Liu Y Tian J Ma J Tang X Rui K. The Long Noncoding RNA IFNG-AS1 Promotes T Helper Type 1 Cells Response in Patients with Hashimoto’s Thyroiditis. Sci Rep (2015) 5:17702. 10.1038/srep17702 26634912 PMC4669486

[288] Papoutsoglou P Tsubakihara Y Caja L Morén A Pallis P Ameur A. The TGFB2-AS1 lncRNA Regulates TGF-β Signaling by Modulating Corepressor Activity. Cell Rep (2019) 28:3182–3198.e11. 10.1016/j.celrep.2019.08.028 31533040 PMC6859500

[289] Li Y Xiang Y Song Y Wan L Yu G Tan L. Dysregulated miR-142, -33b and -423 in granulosa cells target TGFBR1 and SMAD7: a possible role in polycystic ovary syndrome. Mol Hum Reprod (2019) 25:638–46. 10.1093/molehr/gaz014 30865275

[290] Farooqi AA Attar R Qureshi MZ Fayyaz S Sohail MI Sabitaliyevich UY. Interplay of long non-coding RNAs and TGF/SMAD signaling in different cancers. Cell Mol Biol Noisy–Gd Fr (2018) 64:1–6. 10.14715/cmb/2017.64.15.1 30672446

[291] Sun B Liu C Li H Zhang L Luo G Liang S. Research progress on the interactions between long non-coding RNAs and microRNAs in human cancer. Oncol Lett (2020) 19:595–605. 10.3892/ol.2019.11182 31897175 PMC6923957

[292] Du X Liu L Li Q Zhang L Pan Z Li Q. NORFA, long intergenic noncoding RNA, maintains sow fertility by inhibiting granulosa cell death. Commun Biol (2020) 3:131. 10.1038/s42003-020-0864-x 32188888 PMC7080823

[293] Zhang D Tang H-Y Tan L Zhao D-M. MALAT1 is involved in the pathophysiological process of PCOS by modulating TGFβ signaling in granulosa cells. Mol Cell Endocrinol (2020) 499:110589. 10.1016/j.mce.2019.110589 31557499

